# Construction of the novel immune risk scoring system related to CD8^+^ T cells in uterine corpus endometrial carcinoma

**DOI:** 10.1186/s12935-023-02966-y

**Published:** 2023-06-22

**Authors:** Ganghua Zhang, Zhijing Yin, Jianing Fang, Anshan Wu, Guanjun Chen, Ke Cao

**Affiliations:** 1 Department of Oncology, Third Xiangya Hospital, Central South University, Changsha, China; 2 Zhuzhou Hospital Affiliated to Xiangya School of Medicine, Central South University, Zhuzhou, China

**Keywords:** Uterine corpus endometrial carcinoma, CD8^+^ T cell, Immune response, Gene mutation, NIRS

## Abstract

**Background:**

Uterine corpus endometrial carcinoma (UCEC) is a gynecological malignant tumor with high incidence and poor prognosis. Although immunotherapy has brought significant survival benefits to advanced UCEC patients, traditional evaluation indicators cannot accurately identify all potential beneficiaries of immunotherapy. Consequently, it is necessary to construct a new scoring system to predict patient prognosis and responsiveness of immunotherapy.

**Methods:**

CIBERSORT combined with weighted gene co-expression network analysis (WGCNA), non-negative matrix factorization (NMF), and random forest algorithms to screen the module associated with CD8^+^ T cells, and key genes related to prognosis were selected out by univariate, least absolute shrinkage and selection operator (LASSO) and multivariate Cox regression analyses to develop the novel immune risk score (NIRS). Kaplan–Meier (K-M) analysis was used to compare the difference of survival between high- and low- NIRS groups. We  also explored the correlations between NIRS, immune infiltration and immunotherapy, and three external validation sets were used to verify the predictive performance of NIRS. Furthermore, clinical subgroup analysis, mutation analysis, differential expression of immune checkpoints, and drug sensitivity analysis were performed to generate individualized treatments for patients with different risk scores. Finally, gene set variation analysis (GSVA) was conducted to explore the biological functions of NIRS, and qRT-PCR was applied to verify the differential expressions of three trait genes at cellular and tissue levels.

**Results:**

Among the modules clustered by WGCNA, the magenta module was most positively associated with CD8^+^ T cells. Three genes (CTSW, CD3D and CD48) were selected to construct NIRS after multiple screening procedures. NIRS was confirmed as an independent prognostic factor of UCEC, and patients with high NIRS had significantly worse prognosis compared to those with low NIRS. The high NIRS group showed lower levels of infiltrated immune cells, gene mutations, and expression of multiple immune checkpoints, indicating reduced sensitivity to immunotherapy. Three module genes were identified as protective factors positively correlated with the level of CD8^+^ T cells.

**Conclusions:**

In this study, we constructed NIRS as a novel predictive signature of UCEC. NIRS not only differentiates patients with distinct prognoses and immune responsiveness, but also guides their therapeutic regimens.

**Supplementary Information:**

The online version contains supplementary material available at 10.1186/s12935-023-02966-y.

## Introduction

Uterine corpus endometrial carcinoma (UCEC) is an epithelial tumor associated with excessive estrogen secretion, obesity, and Lynch syndrome [1–3]. Among the top 10 cancer categories projected by the American Cancer Society for new cancer cases and deaths in 2023, UCEC ranks fourth in terms of morbidity and sixth in mortality, accounting for seven percent and five percent of female cancers, respectively [4]. Currently, its morbidity and mortality are still rising [3–6]. High incidence and poor prognosis of UCEC pose a significant threat to women’s health. Traditional treatment approaches for UCEC include surgery, chemotherapy, radiotherapy, hormonal therapy, and targeted therapy. However, these treatments have not significantly improved the prognoses of some advanced patients [7]. In recent years, immunotherapy has emerged as a promising avenue for advanced UCEC patients. Combined immunotherapy has shown potential in providing better survival outcomes for advanced patients: Pembrolizumab in combination with Lenvatinib has significantly improved the objective response rate (ORR) and long-term survival in UCEC patients with disease progression [8–11]. In addition to the anti-angiogenic drugs, the combination of immunotherapy and chemotherapy is being explored as well, with Pembrolizumab combined with doxorubicin being shown to exert substantial anti-tumor activity and controllable safety in advanced UCEC patients who did not respond to platinum-based chemotherapy [12]. Thus, immunotherapy is assuming an increasingly important role in the remedy of UCEC.

The remarkable efficacy of immunotherapy in UCEC can be attributed to several factors. Firstly, the tumor immune microenvironment (TIME) of UCEC exhibits an abundance of immune cells, which play a crucial role in tumor development and impact the effectiveness of immunotherapy as well as the prognosis of patients [13–15]. Moreover, there is a high incidence of gene different mismatch repair (dMMR) in UCEC cells. It has been confirmed that tumor subtypes of microsatellite instability-high (MSI-H)/dMMR are correlated with high tumor mutation burden (TMB), which regulates immune infiltration and improves the PD-1/PD-L1 expression levels; thus, improving the efficiency of immunotherapy [16, 17]. Immune checkpoint inhibitors (ICIs) primarily function by blocking inhibitory immune receptors and activating dysfunctional T cells [17–19]. Notably, CD8^+^ T cells, which play a crucial role in adaptive immunity, are actively involved in the process and significantly contribute to the efficacy of immunotherapy as the most influential effectors of the anti-tumor immune response [20, 21]. Numerous studies have demonstrated the significant impact of CD8^+^ T cell infiltration density and activity on the response to immunotherapy in UCEC. Furthermore, the abundance of CD8^+^ T cells is considered as an independent prognostic biomarker for UCEC patients [22–24]. However, there is currently no corresponding criteria to clearly distinguish the patients who would benefit from immunotherapy based on CD8^+^ T cell infiltration levels. In addition, the existing predictive markers cannot accurately identify all patients who would derive benefits from immunotherapy [25, 26]. Hence, it is indispensable to identify biomarkers which can accurately predict the responsiveness of immunotherapy in UCEC patients and their prognoses.

With the rapid advancements in bioinformatics, large medical datasets can now be leveraged to identify molecules with specific functions in the development and progression of UCEC. These molecules can be utilized to construct a novel prediction system that can guide clinical decision-making. As a novel algorithm, weighted gene co-expression network analysis (WGCNA) seeks out highly co-expressed gene modules and identifies the module and genes which are highly correlated with tumors; thus, analyzing gene association patterns among different samples [27–29]. This algorithm has been widely used for various tumors such as lung cancer, bladder cancer, prostate cancer, and breast cancer [30–34]. In this study, we employed WGCNA to investigate the target module and genes associated with immune cells in UCEC. Furthermore, we developed a new immune risk scoring system based on CD8^+^ T cells and demonstrated its important role in forecasting the prognosis of patients and responsiveness of immunotherapy. Lastly, we explored potential correlations between the system and the TIME, immune checkpoints, gene mutation and sensitivities of other anti-tumor therapies.

## Methods

### Data acquisition and arrangement

Combined with the downloaded clinical information, 541 UCEC samples with complete expression profiles and corresponding clinical information were acquired (Table 1). The expression profiles, initially provided in fragments per kilobase million (FPKM) format, were converted into transcripts per million (TPM) format [35], rows and columns with missing values exceeding 50% were removed, and a logarithmic conversion was applied to obtain the final expression profile data in log2(TPM + 1) format. The simple nucleotide variation data of the TCGA-UCEC cohort were obtained from the GDC database in ‘.maf’ format and utilized to calculate the TMB values of each sample. The formula used is as follows: TMB (mut/mb) = total mutation amount (including synonymous, non-synonymous, substitution, insertion, and deletion mutations)/size of target coding area. Downloaded TCGA-UCEC cohort data on four immunophenoscores (IPS) and microsatellite instability (MSI) data from the TCIA database (https://tcia.at). The data of the immunotherapy cohort PRJEB23709 comes from the TIGER database (http://tiger.canceromics.org/), and the immunotherapy cohort IMvigor210 data was derived from the study of Mariathasan et al.[36].

**Table 1:** Baseline Data Sheet for the Cohort of TCGA-UCEC

Characteristic	levels	N (%)
Age	> 65 years old	233 (43.1)
	≤ 65 years old	308 (56.9)
Grade	G1	99 (18.3)
	G2	122 (22.6)
	G3	320 (59.1)
stage	I	336 (62.1)
	II	52 (9.6%)
	III	123 (22.7)
	IV	30 (5.6)
histological_type	Endometrioid endometrial adenocarcinoma	405 (74.9)
	Serous endometrial adenocarcinoma	114 (21.1)
	Mixed serous and endometrioid	22 (4.0)

### Assessment of infiltration level of immune cells in UCEC

CIBERSORT is an immune infiltration analysis software used to evaluate the abundance of immune cells in tumor and normal tissue using the differential expression of marker genes in 22 immune cells [37]. Using the “e1701” R package as the precondition, CIBERSORT was repeated 1000 times to calculate the immune infiltration and filter immune cells with no content in all samples. Samples with P < 0.05 were subsequently screened to determine the immune cell distribution in UCEC immune microenvironment.

### Construction of co-expression network and selection of key module

During the processing of the whole-genome expression profile of the TCGA-UCEC cohort, we filtered out genes with minimal fluctuations, performed sample clustering, and removed outliers to obtain the input gene expression matrix. The gene expression matrix was then integrated with the immune infiltration level results obtained from CIBERSORT, leading to the construction of WGCNA. The “WGCNA” R package was employed to construct a weighted matrix using the Pearson correlation analysis [38]. The PickSoftThreshold function was applied to generate the power scatter plot, and the optimal softpower (β) value was selected according to fitting index and average connectivity. Subsequently, we used the formula AMN =|CMN|β (CMN represents the Pearson correlation value of the paired genes; AMN represents the adjacency relationship between the paired genes; β was the best softpower value) to construct the weighted adjacency matrix. Genes with comparable expression levels were divided into different modules using the dynamic tree cutting method, and hierarchical clustering was carried out in light of the difference degree of topological overlap matrix (TOM) to generate the gene tree. Genes with similar expression profiles were assigned to separate modules, each consisting of at least 60 genes. Determining 0.25 as the threshold of module similarity, we merged similar modules and identified the magenta module (key module) to be highly positively associated with the level of CD8^+^ T cell infiltration.

### Clustering recognition and evaluation based on CD8^+^T Cells

Based on the list of marker genes of immunocytes provided by BindeaG et al.[39], we employed the non-negative matrix factorization (NMF) algorithm to cluster the samples from the UCEC patient cohort according to the gene expression profile of CD8 + T cells [40]. The NMF algorithm used the Brunet method with 10 iterations. We utilized principal component analysis (PCA) and t-distributed stochastic neighbor embedding (tSNE) to achieve dimensionality reduction and visualization of the expression characteristics of CD8^+^T cells among different clusters, and compared the differences of OS among different CD8^+^T cell related clusters by Kaplan–Meier (K-M) survival analysis. Estimation of stromal and immune cells in malignant tumors using expression data (ESTIMATE) algorithm is a method to assess infiltrated mesenchymal and immune cells in various tumor types [41]. The “ESTIMATE” R package was used to calculate the immune microenvironment-related scores for each patient within the TCGA-UCEC cohort, including: StromalScore, ImmuneScore, and ESTIMATEScore. StromalScore indicates the matrix score, ImmuneScore reflects the immune score, and both scores provide insights into the relative infiltration richness of matrix and immune components, respectively, while ESTIMATEScore represents the sum of these two scores, serving as an overall indicator of the immune microenvironment. We performed the differential analysis to compare the differences in these three scores among different subgroups of patients, and conducted CIBERSORT and ssGSEA algorithms to quantify the level of immune cell infiltration in the TIME of UCEC, in which ssGSEA algorithm was implemented using the “GSVA” R package [42].

### Random forest classification and screening of differential genes

The “limma” R package was used to analyze the gene differences between CD8^+^T cells related subtypes in UCEC patients, and finally the differentially expressed genes (DEGs) were obtained by applying log fold change (logFC) ≥ 1, P < 0.05 as the screening criterion (P value was corrected by “FDR” method). Furthermore, the random forest algorithm, implemented through the “randomForest” R package, was used to filter the features of the DEGs [43]. The default number of iterations was set to100, and with the construction of 500 trees, the model was considered to be sufficiently robust. The importance of DEGs was scored using the Gini coefficient method, and genes with a score ≥ 1 were selected as characteristic genes, the screened characteristic DEGs were then intersected with the genes from the WGCNA magenta module and the set of protein-coding genes. Univariate Cox analysis was performed to identify the intersection genes closely related to the prognosis of UCEC patients.

### Construction of the novel immune risk score

After excluding samples with a follow-time of 0, 539 UCEC samples from the TCGA cohort were divided into the training (N = 325) and validation sets (N = 214) in a ratio of 6:4, which allowed for internal validation of the predictive capacities of the novel immune risk score (NIRS). The “glmnet” R package was applied to carry out Least absolute shrinkage and selection operator (LASSO) regression algorithm. LASSO regression is used for dimension reduction and filtering by constructing a penalty function and compressing some coefficients that are set to zero. Finally, using multivariate Cox regression analysis, genes significantly correlated with overall survival (OS) in UCEC patients were further screened to construct the NIRS. The NIRS was calculated using the following formula: NIRS = h_0_t × exp (β_1_x_1_ + β_2_x_2_ + … + β_5_x_5_), where β refers to the regression coefficient, obtained by calculating the inverse natural logarithm exp(β), and h_0_t is the benchmark risk function. All enrolled patients were divided into high NIRS and low NIRS groups according to the median of the risk score. K-M survival analysis was employed to predict and compare OS in both cohorts. Risk factor association plots were utilized to visualize the prognostic landscape of patients in different NIRS groups. Time-dependent receiver operating characteristic (ROC) curve analysis was constructed to evaluate the NIRS prediction accuracy for OS at different time points, and calibration curve was used to test the agreement between the OS predicted by NIRS and the actual OS of patients. Additionally, univariate as well as multivariate Cox regression analyses were carried out, taking into account UCEC common clinical features, to validate the clinical independence of NIRS in predicting OS. Pearson correlation analysis was employed to investigate the interrelationships between the expression levels of pairwise model genes.

### Estimation of immune cell abundance

First of all, we used CIBERSORT and ssGSEA algorithms to evaluate the level of immune cell infiltration in the TIME of UCEC, and compared the landscape of immune infiltration between the high- and low- NIRS groups by differential analysis. Correlation analysis was used to explore the relationship between NIRS and infiltration levels of immunocytes, as well as between CD8^+^T cells infiltration level and model genes. Furthermore, the ESTIMATE algorithm was performed to evaluate and compare the differences in the relative infiltration abundance of matrix components and immune components between patients in high- and low- NIRS groups. This analysis allowed us to gain insights into the overall infiltration patterns of these components within the tumor microenvironment.

### Comparison of immune checkpoints and prediction of immunotherapy effects

To explore the relationship between NIRS and immune checkpoints, we constructed the correlation matrix between NIRS, specific genes and 46 commonly used immune checkpoints. We selected the six most frequently applied immune checkpoints in clinical settings and examined their expression patterns between high- and low- NIRS groups. Next, to assess the responsiveness of patients with different NIRS scores to immune checkpoint inhibitors (ICIs), four IPSs obtained from the TCIA database were used for prediction [44]. The four IPSs were classified as ips_ctla4_pos_pd1_pos, ips_ctla4_neg_pd1_pos, ips_ctla4_pos_pd1_neg, and ips_ctla4_neg_pd1_neg, respectively, which were distinguished by different responses to PD-1 and CTLA-4 inhibitors. Higher IPS values indicate greater sensitivity to the corresponding ICIs. The Tumor Immune Dysfunction and Exclusion (TIDE) database (http://tide.dfci.harvard.edu/) [45] was employed to speculate the function of genes regulating tumor immunity and comprehensively analyze the mechanisms related to tumor immune evasion caused by immune dysfunction and exclusion, so as to effectively predict the therapeutic effects of ICIs. We focused on the tumor immune exclusion score, which is closely related to CD8^+^T cells inhibition, as a predictive index. The expression profile data of patients with high- and low- NIRS were input into TIDE database to analyze the differences in exclusion scores between the two groups. In addition, we employed the submap algorithm [46] available on the GenePattern cloud server to compare the expression profiles of the cohort samples with those of a previously published dataset containing 47 melanoma patients who had responded to immunotherapy [47]. After applying the Bonferroni correction, we explored the correlation between high- and low- NIRS patients and their responses to PD-1 and CTLA-4 inhibitors. Furthermore, we selected two external validation sets, IMvigor210 and PRJEB23709, to verify the predictive performance of NIRS. K-M survival analysis was performed to compare the differences in OS between high- and low- NIRS groups in these two validation cohorts. Additionally, we analyzed the distribution proportions of the four-level efficacy assessments based on the recist1.1 criteria in the patients with high- and low- NIRS, and compared the differences in NIRS scores among the patients with the four efficacy assessed outcomes.

### Mutation analysis based on NIRS

The TMB values for each sample were derived from the gene mutation data in the “.maf” format. All enrolled patients were categorized into high TMB and low TMB groups according to the level of somatic mutations. K-M survival analysis was conducted to investigate the relationship between TMB, in combination with NIRS, and OS of the patients. In addition, differential analysis and Spearman correlation analysis were used to explore the association between NIRS and TMB. In addition, MSI is a mutation-related index that is always used to predict prognosis and immunotherapy efficacy in various tumors including UCEC, gastric cancer, and colorectal cancer. Assessment of MSI mainly relies on four mismatch repair (MMR) proteins: MLH1, MSH2, MSH6 and PMS2 [48]. Differential analysis and Spearman correlation analysis were used to explore potential associations between NIRS and TMB, MSI, and dMMR.

### Clinical subgroup analysis and pharmacotherapy sensitivity analysis

“Stage” and “Grade” are regarded as significant clinical subgroup features closely associated with the prognosis of UCEC. We analyzed the distribution proportion of these clinical subgroup characteristics in high- and low- NIRS groups, and compared the differences in NIRS among patients with different clinical subgroup characteristics using variance analysis, so as to explore the potential relationship between NIRS and common clinical subgroup features. K-M survival analysis was conducted to investigate the prognostic role of NIRS in patients with different Grade and Stage subgroups. Next, based on the Genomics of Drug Sensitivity in Cancer (GDSC) (https://www.cancerrxgene.org/) data source, we constructed the ridge regression model using the “oncoPredict” R package to calculated the predicted half maximal inhibitory concentration (IC_50_) values of commonly used chemotherapeutic agents and targeted therapeutics. The sensitivities of these drugs were reflected by comparing the IC_50_ values in patients with high- and low- NIRS. A lower IC_50_ value indicates greater sensitivity of the patient to the drug.

### Gene set variation analysis

Gene set variation analysis (GSVA) based on the package “GSVA” focused on assessing changes in pathway activity [49], and we obtained four gene sets, “c2.cp.kegg.v7.5.1.symbols.gmt,” “c2.go.v7.5.1.symbols.gmt,” “c2.cp.reactome.v7.5.1. symbols.gmt,” and“h.all.v7.5.1. symbols.gmt”, from the MsigDB database, and explored the significantly different pathways between high- and low- NIRS groups from the four perspectives of the Kyoto Encyclopedia of Genes and Genomes (KEGG) pathway, the Gene Ontology (GO) pathway, the Reactome pathway and the HALLMARK pathway.

### Preparation for UCEC samples and cell lines

The UCEC tissue samples were collected from patients after surgery in the Third Xiangya Hospital of Central South University, China. The histological diagnosis was independently confirmed by two pathologists. All samples were immediately cryopreserved in liquid nitrogen within 30 min. For further analysis, we isolated total RNA from the cancer tissue and performed quantitative reverse transcription polymerase chain reaction (qRT-PCR). The study was conducted in accordance with the approval of the Ethics Committee of the Third Xiangya Hospital of Central South University, China. Informed consent was obtained from all patients involved in the study. Additionally, two human UCEC cell lines, HEC-1A and Ishikawa (ISHI), were purchased from Wuhan Procell Life Technology (Wuhan, China), and Wuhan Pricells Biotechnology & Medicine Co., Ltd (Wuhan, China) provided primary endometrial cells. Details of the culture media used for all cell lines and their respective manufacturers are listed in Additional file 1: Table S1. The media were supplemented with 10% fetal bovine serum (Biosharp Life Sciences, Hefei, China) and 1% penicillin–streptomycin (Biosharp Life Sciences). All cell lines were cultured in the constant temperature incubator at 37 °C and 5% CO_2_, ensuring an environment free from bacteria, yeasts, fungi, or mycoplasma contamination. Finally, siRNA transfection was performed to silence three specific genes in two UCEC cell lines, all RNA inhibitors, including negative control siRNA, were purchased from Tsingke Biotechnology Co., Ltd (Beijing, China). Detailed information regarding the siRNA sequences can be found in Additional file 2: Table S2. HEC-1A and ISHI cell lines were transfected with siRNAs using jetPRIME^®^ siRNA transfection reagent (Polyplus). Subsequent analyses were conducted 24 h post-transfection.

### Quantitative Real-Time RT-PCR

Comparison was performed among HEC-1A, ISHI and the primary endometrial cells, and three UCEC samples with risk scores ranging from low to high were selected for differential expression analysis of model genes based on clinical stage, pathological grade, and histological type determined using pathological reports. Additionally, the expression levels of target genes in HEC-1A cells after transfection with different siRNAs were compared to validate the gene knockdown efficiency of each specific siRNA. Furthermore, the interrelationships among the three specific genes were further confirmed. To further validate the conclusions, ISHI cells transfected with the siRNA demonstrating more pronounced gene knockdown effects were selected, and the expression levels of model genes were explored. Using the RNAex Pro reagent (Accurate Biology) to extract total RNA. Residual genomic DNA contamination was removed from total RNA using 4 × GDNA wiper mix (Vazyme R223-01) and cDNA was reverse transcribed using the 5 × HiScript^®^ II qRT SuperMix II. Finally, ChamQ Universal SYBR qPCR Master Mix (Vazyme#Q711) was used for real-time quantification. GAPDH was selected as internal reference and the 2^−△△ct^ method was applied to standardize the comparative expression levels of target genes. Patient clinical information corresponding to the tissue samples is listed in Table 2 and the primers for qRT-PCR are listed in Additional file 3: Table S3.

**Table 2:** Characteristics of selected UCEC tissue samples

Patients	Age	Tissue origin	Pathology type	Pathological grading	LVSI	FIGO stage
Risk-H	53	Primary Tumor	Adenocarcinoma	G3	+	pIIIb
Risk-M	63	Primary Tumor	Adenocarcinoma	G3	−	pIb
Risk-L	54	Primary Tumor	Adenocarcinoma	G2	−	pIb

G1: well differentiated carcinoma with tumor solid growth area ≤ 5%; G2: moderately differentiated carcinoma with solid growth area accounting for 6–50%; G3: Poorly differentiated carcinoma with solid growth area > 50%. *LVSI* lympho-vascular space invasion, refers to pathologically demonstrated infiltration of a vascular tumor thrombus, including the presence of cancer cells on lymphatic vessels or blood vessel walls. *pIb* The depth of tumor infiltration was ≥ 1/2, and no cervical interstitial invasion was observed. *pIIIb* The tumor spreads locally or regionally, and the vagina or uterus is involved

### Statistical analysis

All bioinformatics statistical analyses in this study were performed using R (version 4.2.1) and Perl languages, among which perl language was mainly used for extensive data cleaning. Unless otherwise specified, differential analysis in this study was carried out via the “limma” R package. The Wilcoxon test was utilized for comparing two groups, while the Kruskal–Wallis test was used for comparisons involving more than two groups. Additionally, correlations were determined using the Spearman correlation analysis. The K-M method and log-rank test were utilized for survival analysis and to compare the difference in prognosis among different groups (by “survival” and “surviminer” R packages). Statistical significance was determined using Graphpad Prism (version 9.0). The differential expressions of HEC-1A, ISHI and primary endometrial cells, as well as the differences in the expression levels of target genes between SiRNA-transfected cells and the negative control group, were analyzed using independent Student’s *t*-test, while one-way ANOVA was applied to compare the difference between UCEC samples. All statistical tests were bilateral, and P-values < 0.05 were considered statistically significant.

## Results

### Immune infiltration composition of samples in TCGA-UCEC cohort

**Fig. 1:**
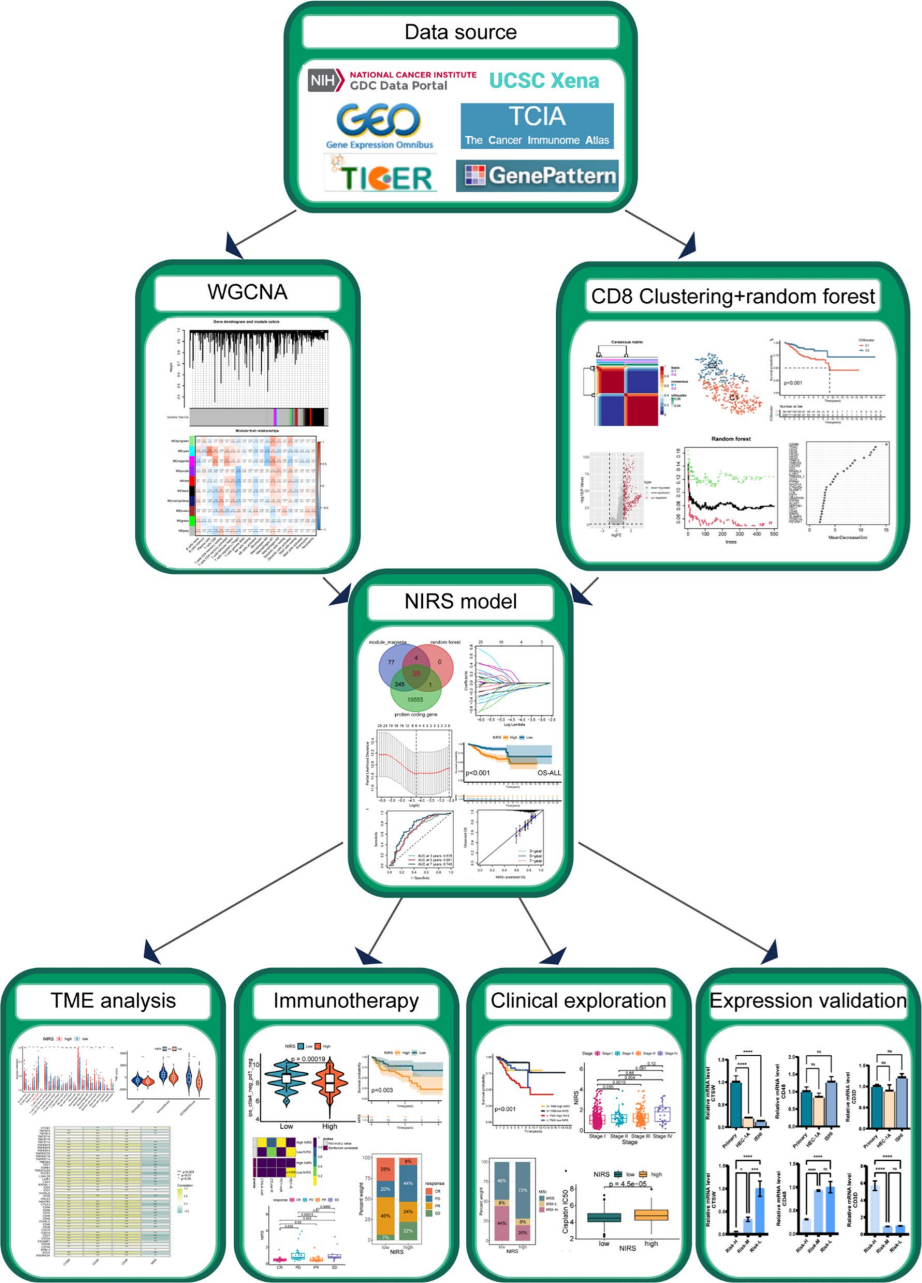
The workflow chart of this study

**Fig. 2:**
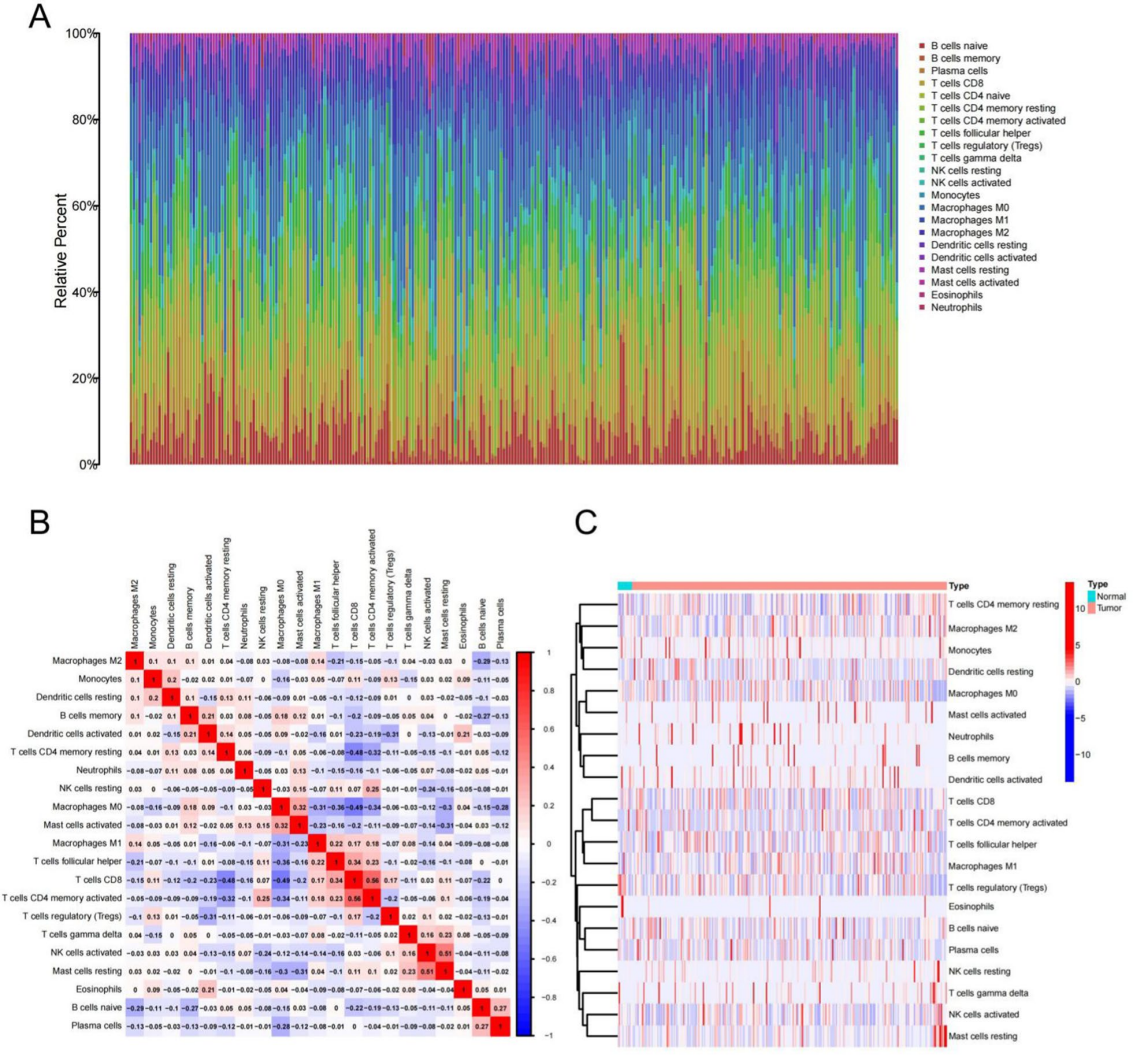
Immune cell infiltration from samples in the TCGA-UCEC cohort. **A** Infiltration distribution of 22 types of immune cells in 544 UCEC samples in the TCGA cohort. **B** Pearson correlation matrix of 21 immune cell infiltration levels. Red is positively correlated, blue is negatively correlated, and the intensity of the color reflects the strength of the correlation. **C** Heatmap of the infiltration of 21 types of immune cells in both normal and UCEC samples. The heatmap utilizes a color gradient, with darker shades of red indicating higher degrees of infiltration, and darker shades of blue indicating lower degrees of infiltration

The workflow of this study is shown in Fig. 1. The CIBERSORT algorithm was performed to assess immune cell infiltration of all samples in the TCGA-UCEC cohort. A bar graph was generated to visualize the landscape of immune infiltration among 22 immunocyte types in 544 UCEC samples (Fig. 2A). We discovered that the infiltration level of “T cells CD4 naïve” was 0 in all UCEC samples, leading us to eliminated them from subsequent analysis. Next, we drew a heatmap to compare the difference in immunological cell infiltration levels between UCEC samples and normal samples (Fig. 2C). Further, we analyzed the correlation between the infiltration levels of 21 types of immunocytes (Fig. 2B), among which CD8^+^ T cell infiltration had the highest positive correlation with active CD4^+^ T cell infiltration (correlation coefficient = 0.56). The infiltration of resting CD4^+^ T cells and macrophage M0 had the highest negative correlation with that of CD8^+^ T cells (coef = -0.48 and -0.49, respectively).

### Screening of related genes in CD8^+^ T cells based on WGCNA

**Fig. 3:**
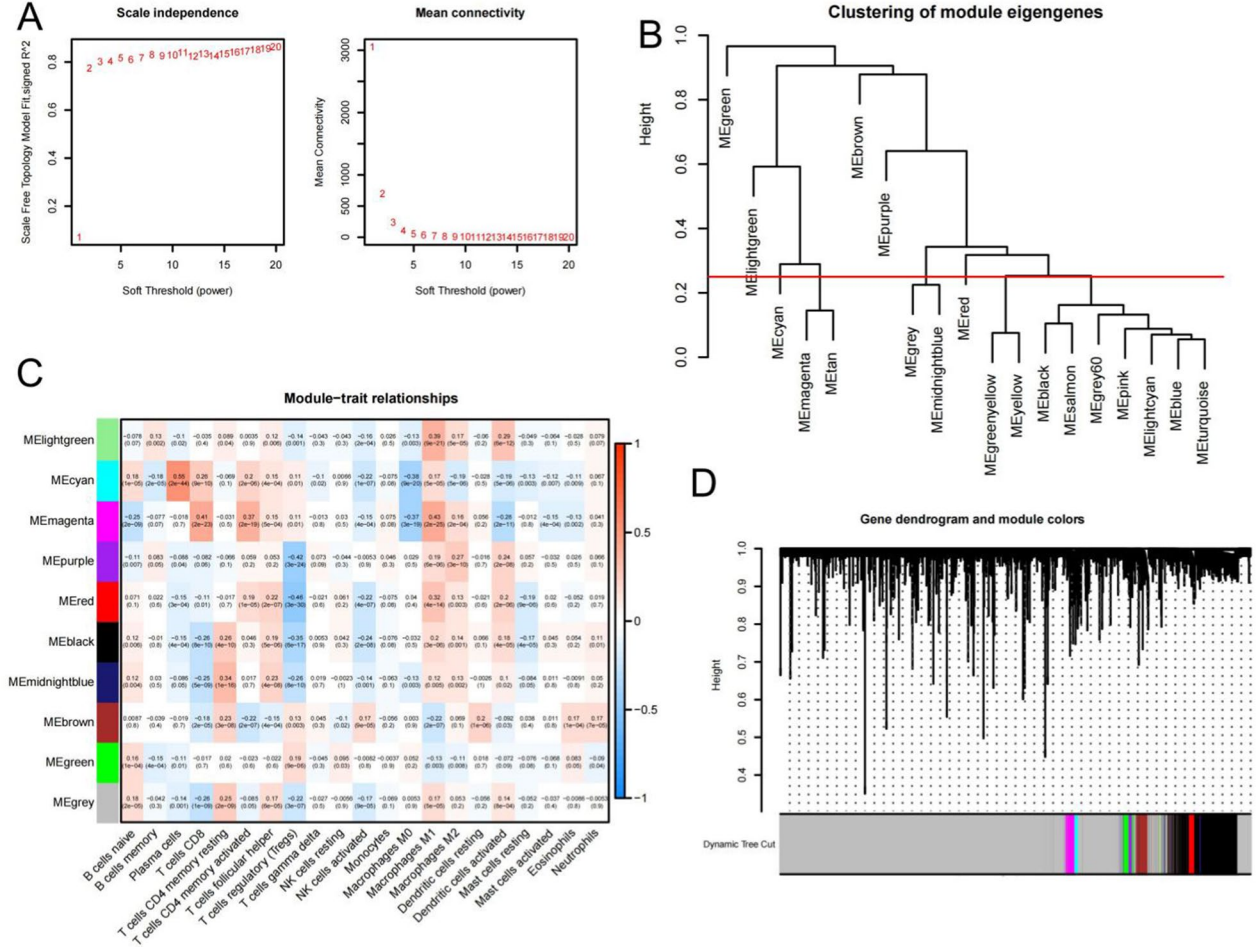
Identification of modules associated with CD8 + T cells in the UCEC cohort.** A** Topological analysis of soft threshold parameters. The optimal soft threshold was determined to be 18 by assessing the fitting index and average connectivity. **B** Cluster dendrogram of module eigengenes. 19 gene modules were clustered and modules with high similarity were combined, resulting in the identification of 10 distinct modules. **C** Heatmap illustrating the correlation between the 10 gene modules and 21 types of immune infiltrating cells. The numbers in each square represent the Pearson correlation coefficients, with red indicating a positive correlation, blue indicating a negative correlation, and the darkness of the color representing the strength of the correlation. The magenta module showed the highest positive correlation with CD8^+^T cells. **D** Cluster dendrogram of the 10 gene modules based on 56719 gene expression profiles in the TCGA-UCEC cohort

We performed WGCNA based on UCEC expression profiles containing 56,719 genes to identify genes closely related to immune cells. The 544 UCEC samples were clustered and any outliers were removed to generate the dendrogram of the samples. According to the trend of fitted index and average connectivity, softpower (β) = 18 (scalefreeR^2^ = 0.8510) was determined as the optimal soft threshold (Fig. 3A), the gene correlation matrix was obtained, and genes with similar expressions were grouped together to form a module. The dynamic clipping module identification (Fig. 3D) was carried out, the hierarchical clustering tree was constructed, and 19 modules were formed (Fig. 3B). The modules with high similarity were combined to obtain 10 modules (Fig. 3D). Further generated the heatmap of the relationship between the 10 modules and 21 immunocytes, and discovered that magenta modules had the highest association with CD8^+^ T cells. In addition, magenta modules had higher correlation with M1 macrophage than other modules (Fig. 3C). The magenta module (464 genes) was selected for further analysis.

### Unsupervised clustering and subtype identification of CD8^+^T cells

**Fig. 4:**
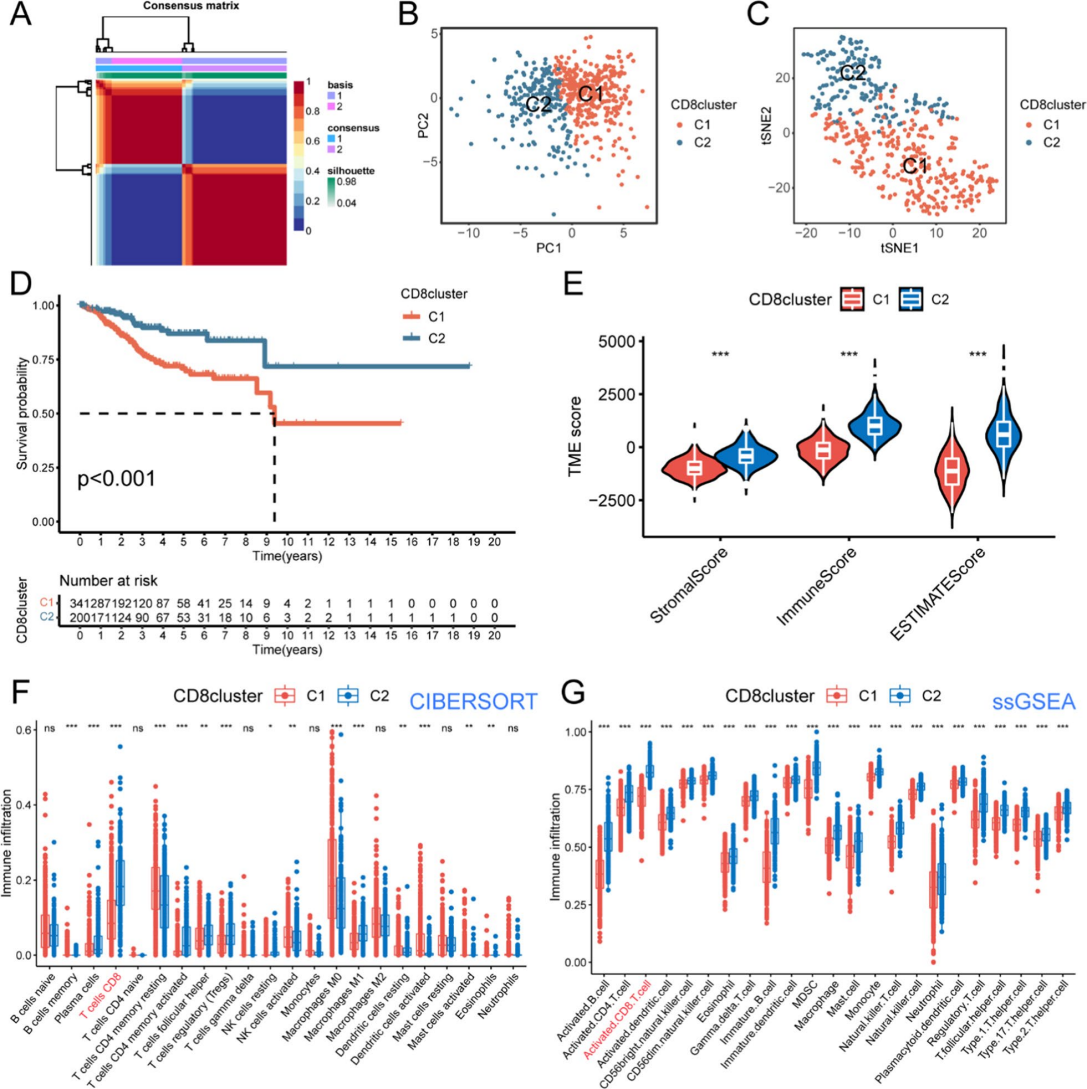
Construction of subgroups based on CD8^+^T cells and exploration of survival and immune characteristics.** A** Non-negative matrix factorization (NMF) was employed to partition the TCGA-UCEC cohort samples into two distinct subtypes, C1 and C2 (k = 2). **B**, **C** Dimensionality reduction using PCA and tSNE algorithms revealed significant dispartities in CD8^+^T cell-based gene expression profiles between the two subtypes **D** K-M survival curves of UCEC patients with CD8^+^T cell molecular subtypes in the two groups demonstrated a noteworthy increase in OS among patients belonging to group C2 compared to shoes in group C1. **E** Comparison of the stromal score, immune score and ESTIMATE score between patients in the C1 and C2 subgroups. **F**, **G** Box plots on CIBERSORT and ssGSEA algorithms illustrated immune infiltration landscapes in the TIME of patients in C1 and C2 groups, in which the infiltration level of CD8.^+^T cells showed significant differences between the two algorithms. ns: no significant statistical difference, *P < 0.05, **P < 0.01, ***P < 0.001

We performed NMF clustering and subtype identification based on the gene expression profile of CD8^+^T cells in the TCGA-UCEC cohort. It was found that the silhouette coefficient reached the maximum when K = 2, indicating the presence of two distinct clusters, designated as C1 and C2 (Fig. 4A). Additionally, a significant variation in the cophenetic coefficient was observed when K = 3, further supporting the division of samples into two clusters. The dimensionality reduction of two subtypes of patients by PCA and tSNE algorithms showed the distinguishability of their characteristics of gene expression profile marked by CD8^+^T cells (Fig. 4B, C). Moreover, there was a significant difference in OS between the two groups. K-M survival analysis manifested that patients in group C2 exhibited significantly better prognosis than those in group C1 (P < 0.001, Fig. 4D). Furthermore, we performed the ESTIMATE package in R to quantify the TIME of the two subtypes, and the matrix, immune, and comprehensive ESTIMATE scores were significantly higher in group C2 compared to group C1 (P < 0.001, Fig. 4E). Finally, the abundance of immune cell infiltration in the TIME was evaluated by CIBERSORT and ssGSEA algorithms, and the results indicated a significant difference in the landscape of immune infiltration between the two subtypes. Notably, the infiltration level of CD8^+^T cells in group C2 was significantly higher than that in group C1 (P < 0.001, Fig. 4F, G).

### The secondary screening of DEGs in random forest

**Fig. 5:**
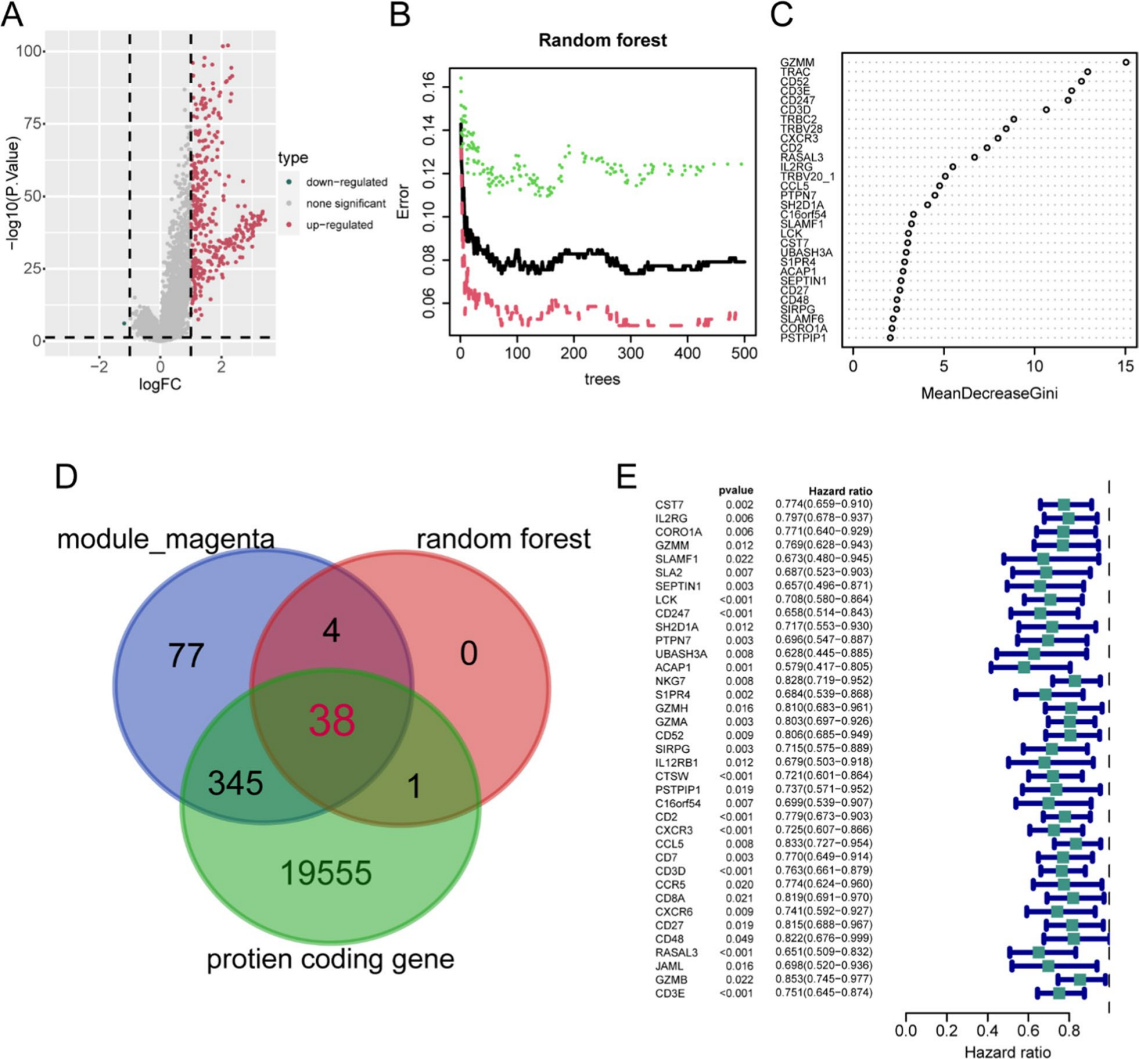
Secondary classification and screening of differentially expressed genes (DEGs).** A** Volcano plot depicting the DEGs between two groups of CD8^+^ T cell molecular subtypes (C2vs.C1). **B** Random forest analysis identified 370 significantly upregulated DEGs in the C2 subtype. The green line represents the error in the experimental group (C2 subtype), the red line was the error in the control group (C1 subtype), and the error of all samples was represented in black. **C** Following random forest screening, a total of 43 DEGs were identified between the C1 and C2 subtypes. **D** Venn diagram illustrating the intersection of the 43 characteristic genes with WGCNA’s magenta module genes and protein coding gene sets, resulting in a total of 38 overlapping genes. **E** Forest plot presenting the result of univariate Cox regression analysis for genes associated with the prognosis of UCEC, revealing 37 protective genes

To identify key molecules influencing the immunological features and prognosis of UCEC patients, we analyzed the differences in gene expression profiles between the two patient subtypes, the volcano plot revealed a set of significantly up-regulated and down-regulated genes of group C2 when compared to group C1 (Fig. 5A). Subsequently, we selected 370 DEGs between the two clusters for random forest feature filtering. When 116 trees were established, the error of all samples reached the minimum (Fig. 5B). Importance ratings were assigned to DEGs based on the Gini coefficient, and genes with a score above 1 were selected as disease-characteristic genes (Fig. 5C). To further identify genes specifically associated with CD8^+^ T cells in UCEC, we intersected the 43 screened DEGs with WGCNA magenta module genes, as well as the set of protein-coding genes. Consequently, we obtain 38 relevant genes (Fig. 5D). Furthermore, univariate Cox regression analysis was performed on these 38 genes, and 37 genes were found to be closely related to OS in UCEC patients (P < 0.05, Fig. 5E).

### Identification of system genes and construction of NIRS

**Fig. 6:**
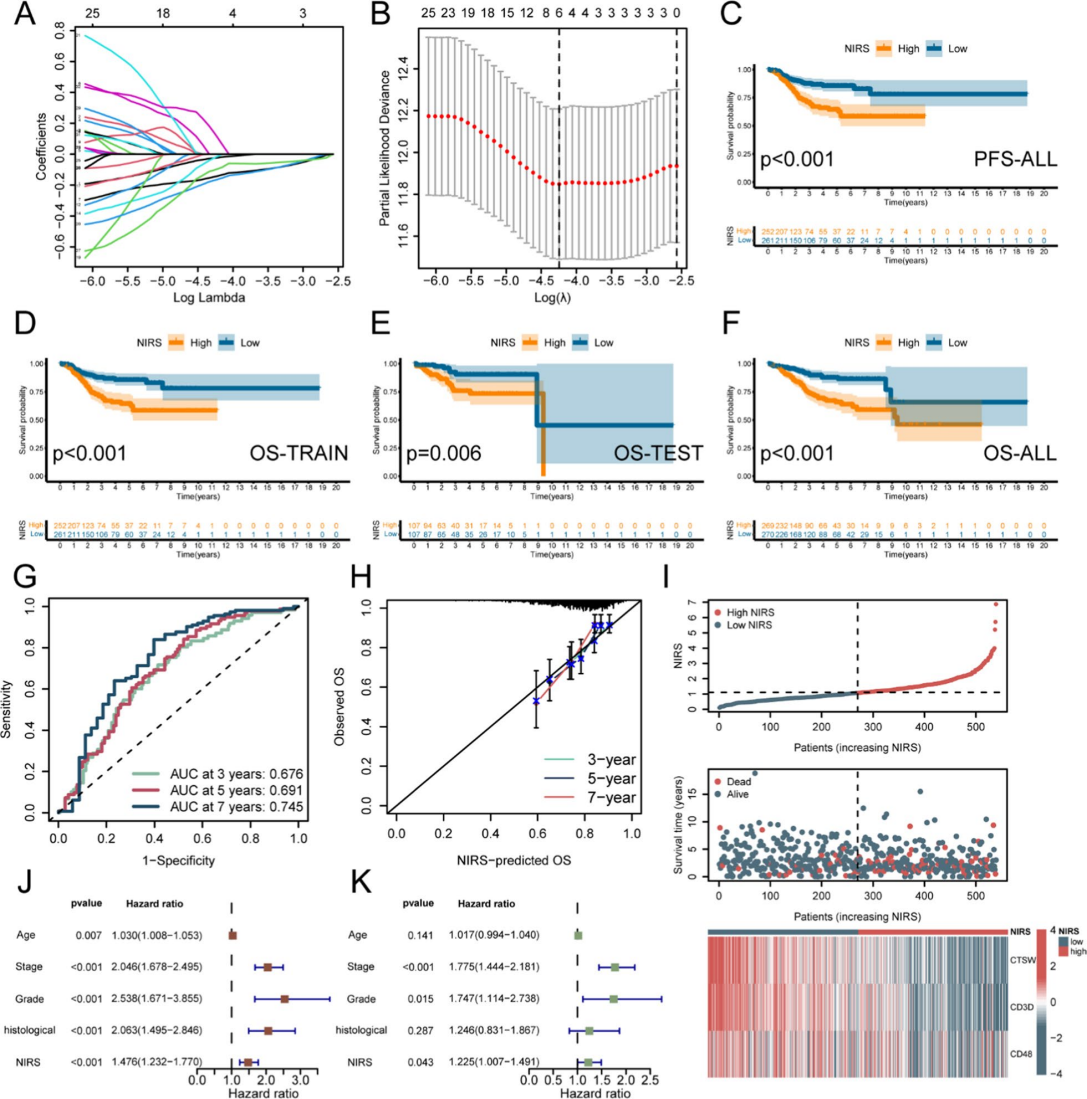
Constuction of novel immune risk score (NIRS).** A** Ten-fold cross-validation of the coefficients of 37 protective genes in the LASSO regression model. **B** Robustness test of the LASSO model with varying number of protective genes revealed the highest stability when the number was 6, and a total of six characteristic genes were obtained. **C** K-M survival curve of patients with high- and low- NIRS in the TCGA-UCEC cohort, and the ordinate represented the progression-free survival (PFS). **D**–**F** K-M survival curves of patients with high- and low- NIRS in the training set, validation set and the overall cohort, and the ordinate represented the overall survival (OS). **G**, **H** Time-dependent ROC curve analysis and calibration curve of NIRS at 3-, 5-, and 7-year intervals. **I** Distribution plot, survival scatter plot and expression profiles of model genes in patients with high- and low- NIRS in the cohort. Red represented high expression and green represented low expression. **J**, **K** Univariate and multivariate Cox regression analyses of NIRS and key prognostic clinical features of UCEC

Furthermore, we conducted LASSO regression analysis of these 37 genes for feature screening and selected six genes for further analysis (Fig. 3C, D). Subsequently, through multivariate Cox regression analysis, we identified three key genes and constructed the NIRS. Based on the median NIRS value as a cutoff, patients were separated into high- and low- NIRS groups. The risk factor correlation chart visualized the survival distribution of the enrolled patients according to increasing NIRS scores, indicating higher mortality in the high NIRS group. The heatmap displayed significant differences in the expression patterns of the three system genes between the high- and low- NIRS groups, with all three genes showing high expression in the low NIRS group (Fig. 6I). According to the K-M survival analysis in the training set, we found that the prognosis of patients in the high NIRS group was significantly worse than that in the low NIRS group (P < 0.001, Fig. 6D), and this finding was validated in the validation set (P = 0.006, Fig. 6E). Analyzing the entire cohort, patients in the low NIRS group achieved longer OS and PFS (P < 0.001, Fig. 6C, 6F). The area under curve (AUC) was used to reflect the predictive efficacy of clinical factors and NIRS, the result showed that the AUC values of NIRS for years 3-, 5-, and 7-are 0.676, 0.691, and 0.745, respectively, and the predictive ability increased with longer time points (Fig. 6G). The calibration curve showed that the OS predicted by NIRS had a high concordance with the actual situation (Fig. 6H). Finally, univariate (Fig. 6J) and multivariate (Fig. 6K) Cox regression analyses confirmed that stage (P < 0.001), grade (P = 0.015), and NIRS (P = 0.043) were independent factors affecting the prognosis of UCEC.

### Analyses of immune microenvironment of UCEC based on NIRS

**Fig. 7:**
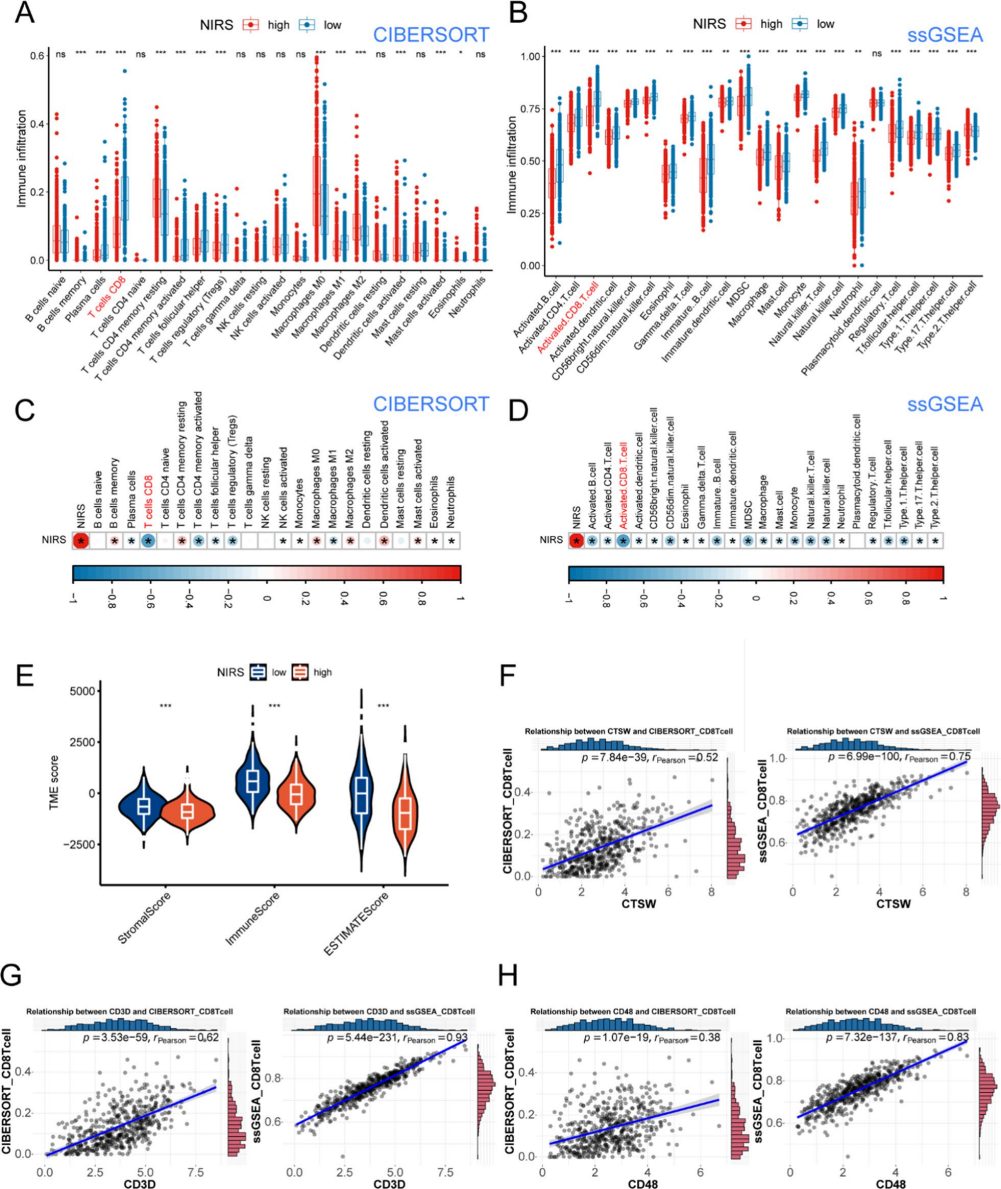
Correlation analyses between NIRS and immune cell infiltration. **A**, **B** Box plots based on CIBERSORT and ssGSEA algorithms depicted the infiltration landscape of 22 and 23 immune cells in the TIME of patients with high- and low- NIRS, and the infiltration levels of CD8^+^ T cells showed significant differences in both algorithms. **C**, **D** The correlation matrix between NIRS and 22 and 23 kinds of immune cell infiltration levels were obtained based on CIBERSORT and ssGSEA algorithms. Red represented positive correlation, blue represented negative correlation, in which CD8^+^ T cells showed the strongest negative correlation with NIRS in both algorithms. **E** The differences of the stromal, immune and ESTIMATE scores between high- and low- NIRS groups. **F–H** Correlation analyses of characteristic genes CTSW (**F**), CD3D (**G**) and CD48 (**H**) with CD8^+^T cell infiltration levels assessed by CIBERSORT and ssGSEA algorithms. *ns* no significant difference, *P < 0.05, **P < 0.01, ***P < 0.001

To explore the potential relationship between NIRS and UCEC immune microenvironment, we performed CIBERSORT and ssGSEA algorithms to evaluate the abundance of immune cell infiltration in the samples. Box plots generated by the two algorithms showed that the level of CD8^+^T cell infiltration in the low NIRS group was significantly higher than that in the high NIRS group (Fig. 7A, B). Correlation analysis was used to explore the tightness of association between NIRS and immune cell infiltration, which revealed that there was the strongest negative correlation between NIRS and CD8^+^T cells (Fig. 7C, D). Subsequently, we employed the ESTIMATE R package to score the TIME. The matrix, immune and comprehensive ESTIMATE scores were significantly higher in patients with low NIRS compared to those with high NIRS (Fig. 7E), suggesting that the stromal and immune components in the TIME of the low NIRS group were more abundant than those in the high NIRS group. Moreover, we used CIBERSORT and ssGSEA algorithms to explore the correlation between the three system genes and the infiltration of CD8^+^T cells. The results showed that CTSW (R_CIBERSORT_ = 0.52; R_ssGSEA_ = 0.75, P < 0.001, Fig. 7F), CD48 (R_CIBERSORT_ = 0.62; R_ssGSEA_ = 0.93, P < 0.001, Fig. 7G) and CD3D (R_CIBERSORT_ = 0.38; R_ssGSEA_ = 0.83, P < 0.001, Fig. 7H) were positively correlated with the infiltration level of CD8^+^T cells.

### Exploration of related checkpoints and efficacy of immunotherapy based on NIRS

**Fig. 8:**
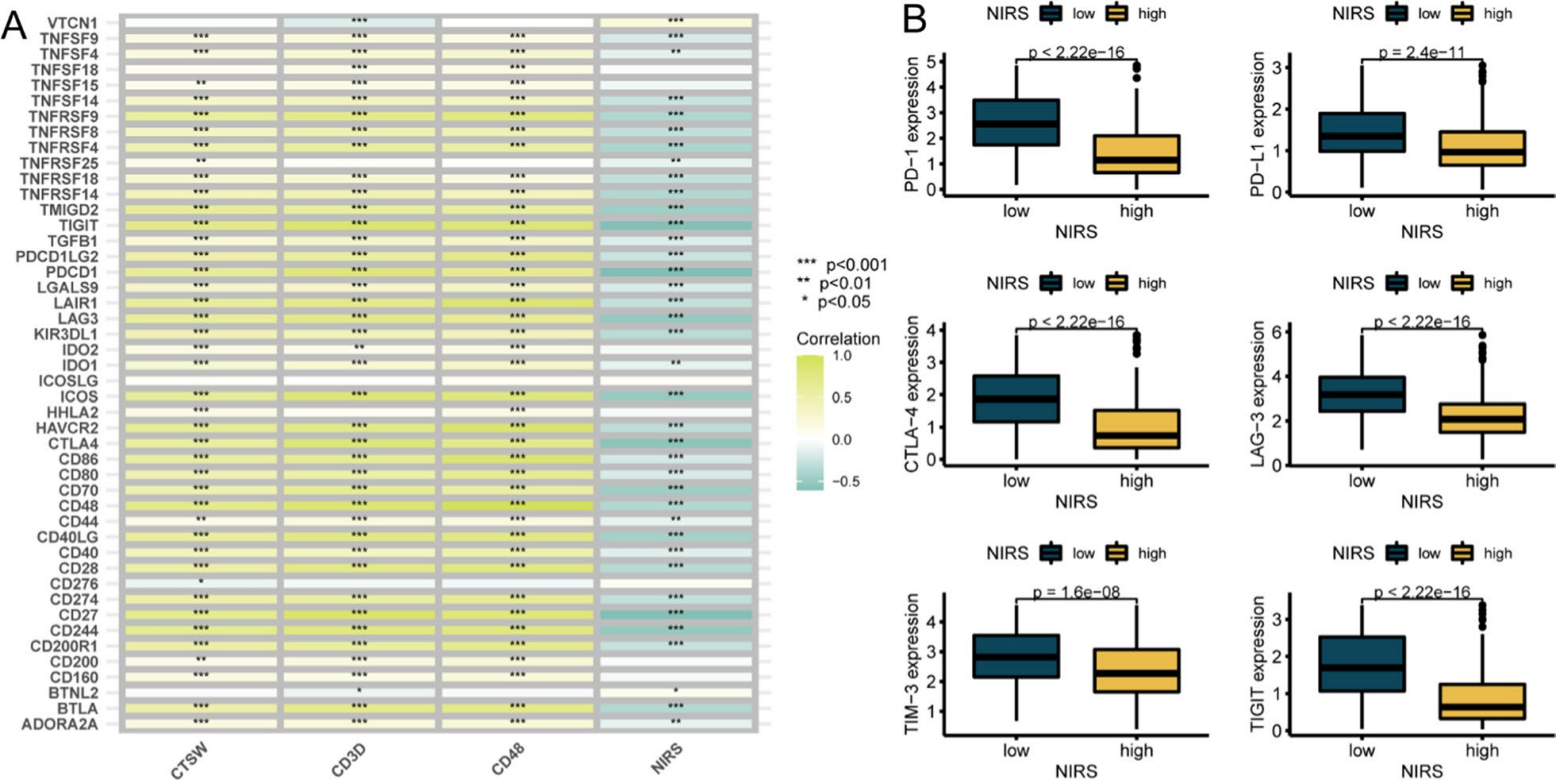
Correlation analyses between NIRS and immune checkpoints.** A** The correlation matrix between 46 immune checkpoints with three system genes and NIRS expression. Yellow represented positive correlation and green represented negative correlation. **B** Box plots of differential expression levels of six immune checkpoints PD-1, PD-L1, CTLA-4, LAG-3, TIM-3, and TIGIT in patients with high- and low- NIRS groups

**Fig. 9:**
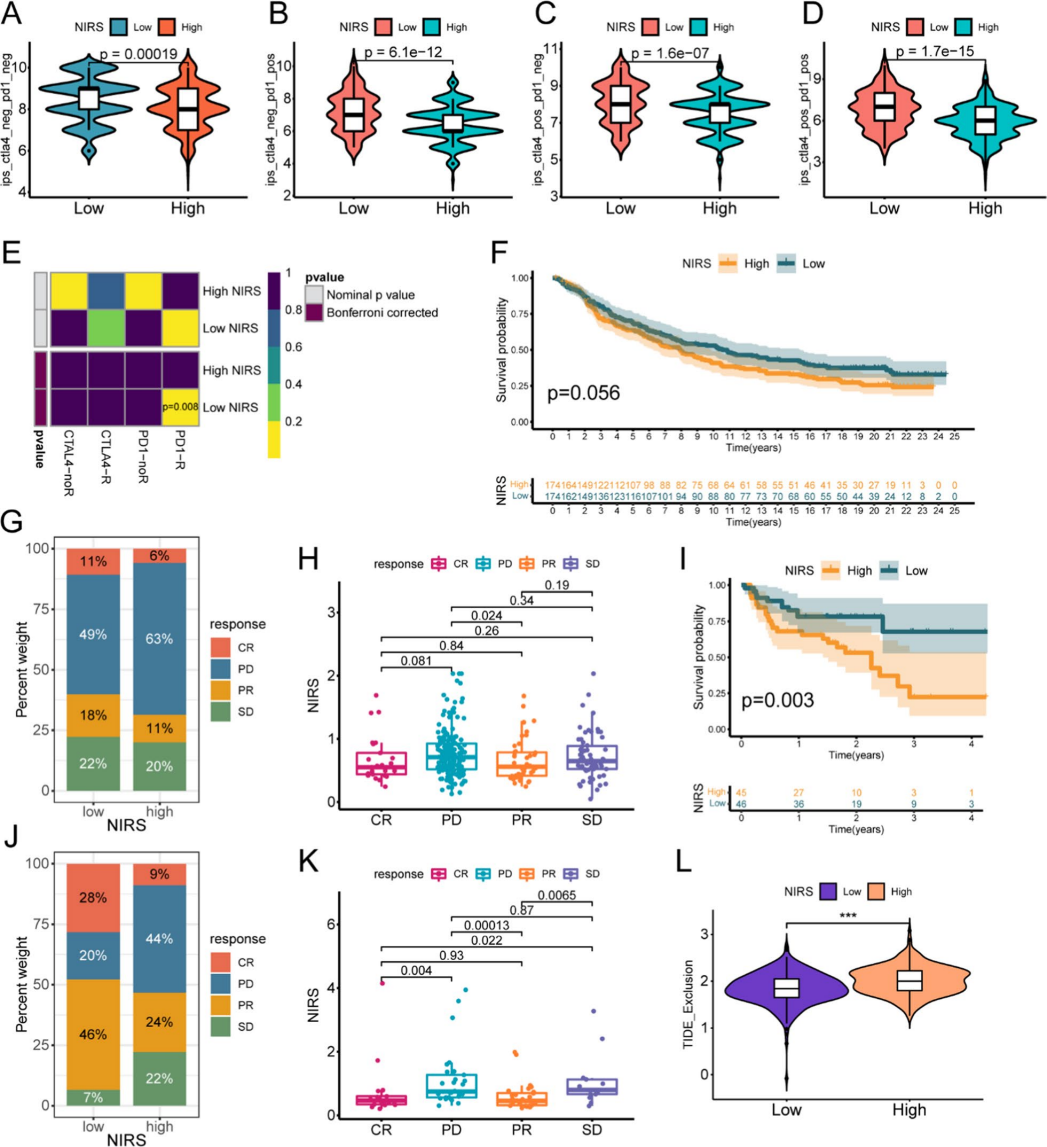
The ability of NIRS to predict the responsiveness of immunotherapy and its external validation. **A-D** Analyses of the curative effect of four immune checkpoint inhibitors (ICIs) in high- and low- NIRS groups. **E** The external validation of the efficacy of NIRS in predicting the responsiveness of PD-1 and CTLA-4 inhibitors, R indicated sensitivity to ICIs of the corresponding checkpoint, and noR indicated insensitivity. Low NIRS group was significantly associated with PD-1 inhibitor responsiveness after Bonferroni correction. **F**–**H** K-M survival curve of patients with high- and low- NIRS (**F**), the distribution of the four-level curative effect evaluation indicators in the high- and low- NIRS groups after immunotherapy (**G**) and the box plot of the difference between four curative effect outcomes and NIRS (**H**) in the IMvigor210 cohort. **I**–**K** K-M survival curve of patients with high- and low- NIRS (**I**), the distribution of the four-level curative effect evaluation indicators in the high- and low- NIRS groups after immunotherapy (**J**) and the box plot of the difference between four curative effect outcomes and NIRS (**K**) in the PRJEB23709 cohort. **L** Differential analysis of immune exclusion between high- and low- NIRS groups, ***P < 0.001

To explore the potential relationship between NIRS and immunotherapy, we constructed a correlation matrix between the three system genes, NIRS, and 46 common immune checkpoints (Fig. 8A). The expression levels of most immune checkpoints were positively correlated with the expression of three system genes, while negatively correlated with the expression of NIRS. Furthermore, we compared the expression levels of the six most commonly studied checkpoints in clinical trials, PD-1, PD-L1, CTLA-4, LAG-3, TIM-3 and TIGIT between patients with the high- and low- NIRS groups. Box plots demonstrated that the expression levels of above targets in patients with low NIRS were significantly higher than those in patients with high NIRS (Fig. 8B). In addition, we analyzed the differences in the responsiveness of patients in different NIRS groups to PD-1 and CTLA-4 inhibitors based on the TCIA database. The results showed that the four IPS scores of patients in the low NIRS group were significantly higher than those in the high NIRS group (P < 0.001, Fig. 9A–D), indicating that the responsiveness of patients in the low NIRS group to PD-1 and CTLA-4 inhibitors was higher than that in the high NIRS group. Meanwhile, the probability of tumor immune exclusion in the high NIRS group was significantly higher than that in the low NIRS group, as indicated by the TIDE database (P < 0.001, Fig. 9L). To further validate the predictive efficacy of NIRS on immunotherapy outcomes, we included external cohorts for analysis. Firstly, the submap algorithm was used to predict the correlation between high- and low- NIRS patients and the response to immunotherapy based on a published cohort. After Bonferroni correction, a significant positive correlation was observed between patients with low NIRS and the response to PD-1 inhibitors (P = 0.008, Fig. 9E). Subsequently, we downloaded the data from the IMvigor210 cohort, comprising 348 patients with bladder cancer treated with Atezolizumab and 91 samples from the PRJEB2309 cohort with melanoma treated with PD-1 inhibitors, for prognostic analysis. We found that in the IMvigor210 validation set, patients with low NIRS displayed a better trend towards improved OS compared to those with high NIRS (P = 0.056, Fig. 9F), although the result did not reach statistical significance. The proportion of progression after receiving Atezolizumab in the high NIRS group was significantly higher than that in the low NIRS group (63% vs.49%, Fig. 9J), and the four efficacy outcomes evaluated based on recist1.1 criteria showed that patients with progressive (PD) disease had significantly higher NIRS scores than those with partial response (PR) (P = 0.024, Fig. 9H). Similarly, in the external validation of the PRJEB2309 cohort, patients in the high NIRS group not only had worse prognoses (P = 0.003, Fig. 9I), but also had a significantly lower proportion of objective remission than those in the low NIRS group after treatment with PD-1 inhibitors (33% vs.74%, Fig. 9J). The box plot displayed that the NIRS scores of patients with complete response (CR) and PR were significantly lower than those with stable disease (SD) and PD (P < 0.05, Fig. 9K).

### Mutation analysis based on NIRS

**Fig. 10:**
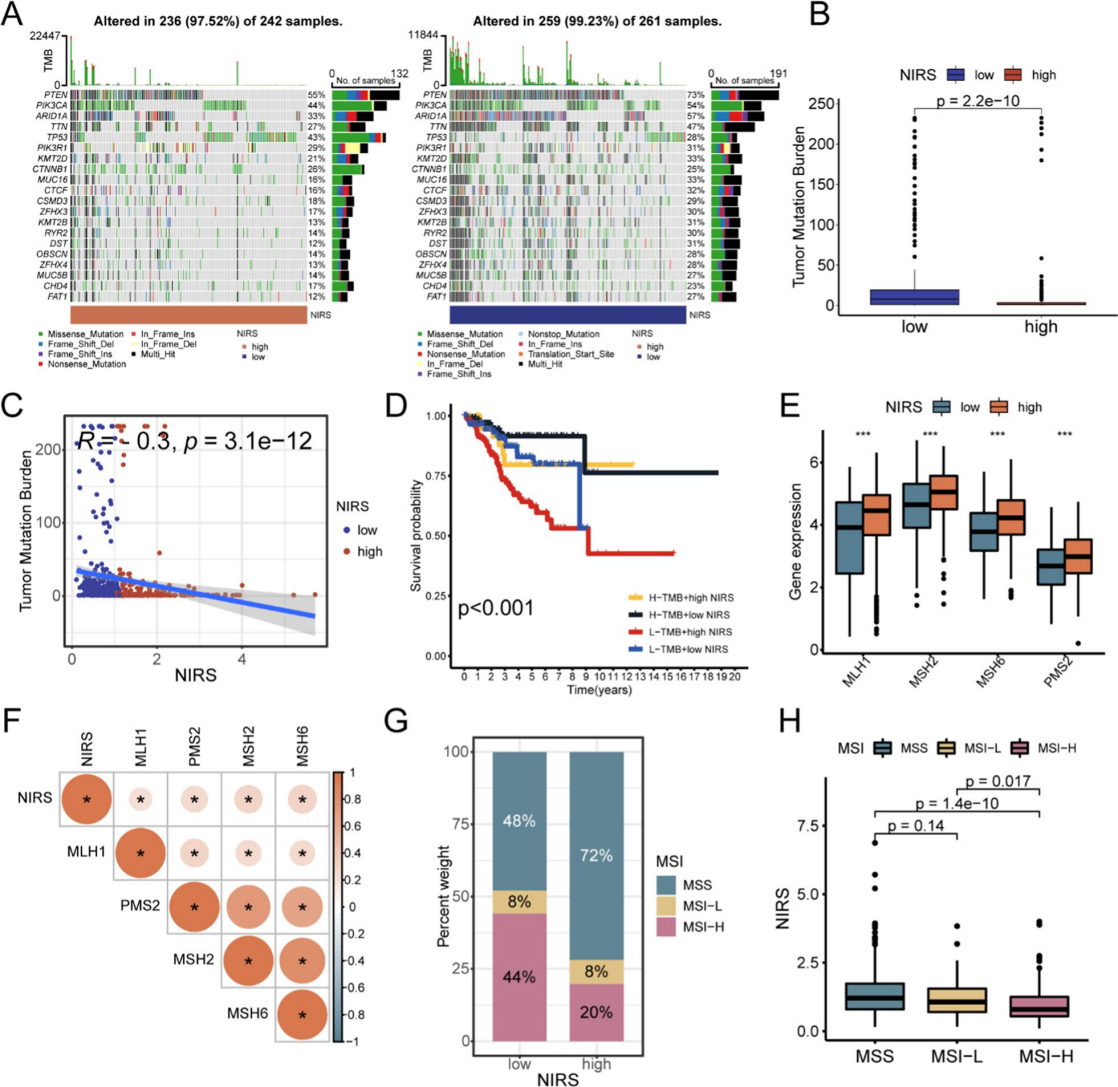
Exploration of the potential correlation between NIRS and mutations.** A** The landscape of gene mutations in patients with high- and low- NIRS. **B** Differences in TMB between patients with high- and low- NIRS. **C** Correlation analysis between TMB and NIRS indicated that with the increase of NIRS, TMB showed a downward trend. **D** K-M survival analysis between patients with different TMB and NIRS status. **E** Differential expression of four mismatch repair (MMR) proteins in patients with high- and low- NIRS. **F** Correlation matrix between NIRS and the expression levels of four MMR proteins. Red represented positive correlation and blue represented negative correlation. **G** Distribution proportion of three microsatellite states in patients with high- and low- NIRS. **H** Differential analysis of NIRS scores in patients with three kinds of microsatellite status

To explore the variations in gene mutations among patients in different NIRS groups, we mapped the gene mutations between the two groups (Fig. 10A). We observed a higher frequency of PTEN mutation in patients with low NIRS, while the frequency of TP53 mutation was lower than that in patients with high NIRS. TMB is an important indicator to predict the efficacy of immunotherapy, with higher TMB generally associated with better immunotherapy outcomes. The potential correlation between NIRS and TMB was explored, it showed that the TMB value of high NIRS group was significantly lower than that of low NIRS group (P < 0.001, Fig. 10B), and there was a significant negative correlation between NIRS and TMB (R = − 0.3, P < 0.011, Fig. 10C). Combining NIRS and TMB predictions, our comprehensive prognosis comparison showed that patients in the high TMB + low NIRS group had the highest OS among the four groups, while the low TMB + high NIRS group had the lowest OS (P < 0.001, Fig. 10D). MSI status is one of the most definite markers for evaluating the efficacy of immunotherapy in UCEC patients, and patients with MSI-H are generally considered to be more responsive to immunotherapy. MSI is determined by the expression of four MMR proteins, and lower expression levels indicate higher MSI. Differential analysis based on the expression of MMR proteins showed that the expression levels of MLH1,MSH2,MSH6 and PMS2 in the low NIRS group were significantly lower than those in the high NIRS group (P < 0.001, Fig. 10E), and there was a positive correlation between NIRS and the expression of four MMR proteins (Fig. 10F). Based on the TCIA database, we analyzed the correlation between MSI status and NIRS in the TCGA-UCEC cohort, and found that the proportion of MSS cases in the high NIRS group was higher than that in the low NIRS group, while the proportion of MSI-H cases was lower than that in the low NIRS group (Fig. 10G). The box plot revealed that the comprehensive NIRS score was the highest in the MSS group, while the MSI-H group had the lowest overall NIRS score, with a significant difference between the two groups (P < 0.001, Fig. 10H).

### Clinical subgroup analysis and antineoplastic susceptibility analysis of NIRS

**Fig. 11:**
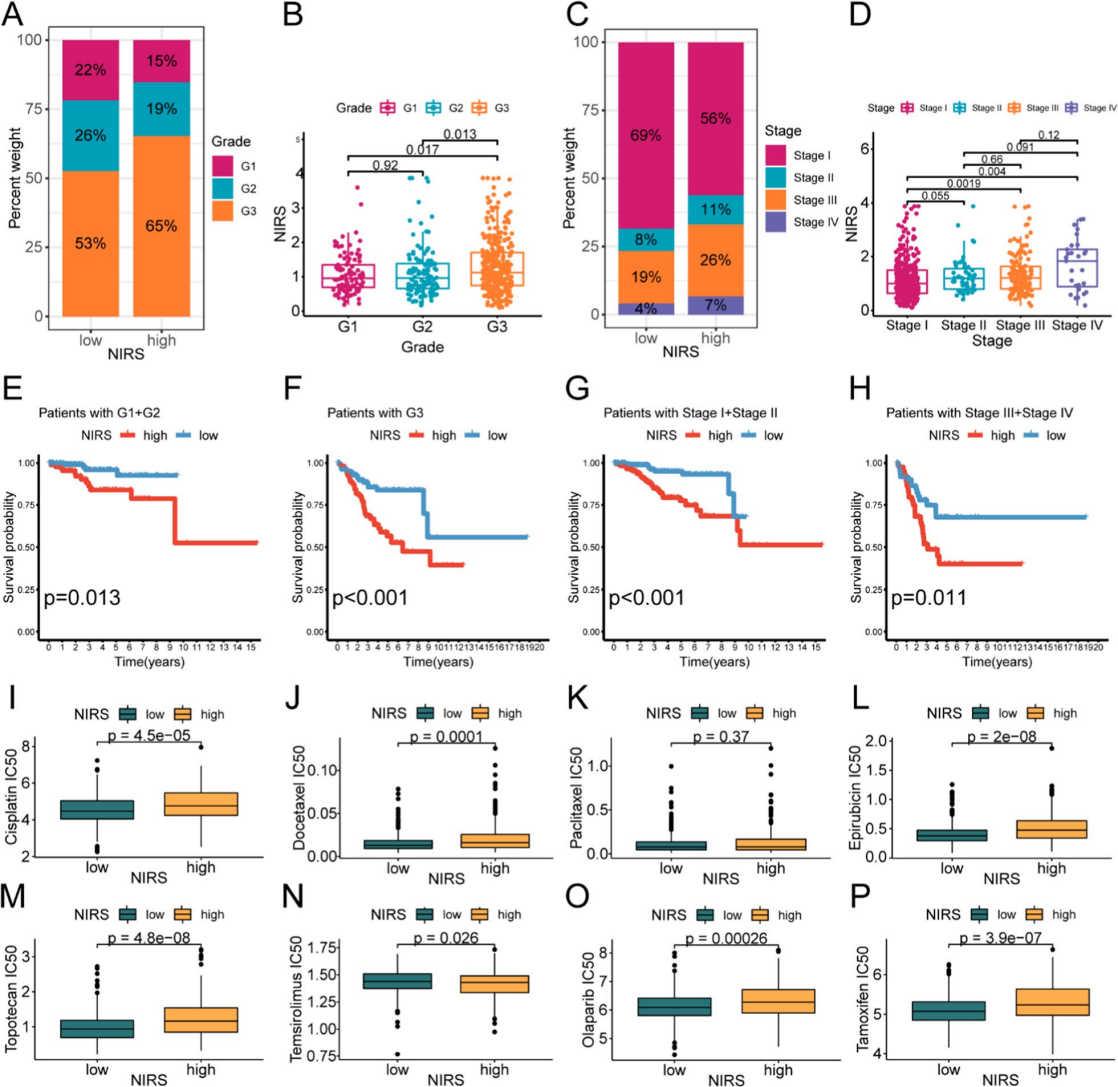
Association between NIRS, clinical prognostic features and treatment sensitivity in UCEC. **A**, **B** The distribution proportion of three pathological grades in patients with high- and low- NIRS and the difference of NIRS scores in patients with different pathological grades. **C**, **D** The distribution proportion of four clinical stages in patients with high- and low- NIRS and the difference of NIRS scores in patients with different clinical stages. **E–H** K-M survival analyses of patients with high- and low- NIRS in different pathological grades and clinical stages. (**I–P**) Drug susceptibility analyses of different NIRS groups, the predicted IC50 values of cisplatin **(I)**, docetaxel **(J)**, paclitaxel (**K**), epirubicin (**L**), topotecan (**M**), temsirolimus (**N**), olaparib (**O**) and tamoxifen (**P**) were compared between the two groups

To explore the potential association between NIRS and important clinical prognostic features, we assessed the distribution proportion of clinical stage and pathological grade between the high- and low- NIRS groups and visualized the result using bar plots. The analysis revealed that the proportion of patients with G3 and III-IV stage in the high NIRS group was higher than that in the low NIRS group (Fig. 11A, C). Moreover, patients with G3 grade had significantly higher NIRS scores compared to those with G1 and G2 (Fig. 11B), and the NIRS scores of patients in stage III and stage IV were significantly higher than those in stage I (Fig. 11D). In addition to immunotherapy, conventional chemotherapy, targeted therapy and endocrine therapy are common treatment options for UCEC as well. Based on the oncoPredict algorithm, we further predicted the sensitivities of these treatments in patients with different NIRS groups (Fig. 11I–P). Box plots revealed that patients with low NIRS were more sensitive to cisplatin (P < 0.00001), docetaxel (P < 0.0001), topotecan (P < 0.00001), olaparib (P = 0.00026) and tamoxifen (P < 0.00001), while the high NIRS group exhibited higher sensitivity to tesirolimus (P < 0.026). For paclitaxel (P = 0.37), there was no significant difference in predicted drug sensitivity between the two groups.

### GSVA analyses related to NIRS

**Fig. 12:**
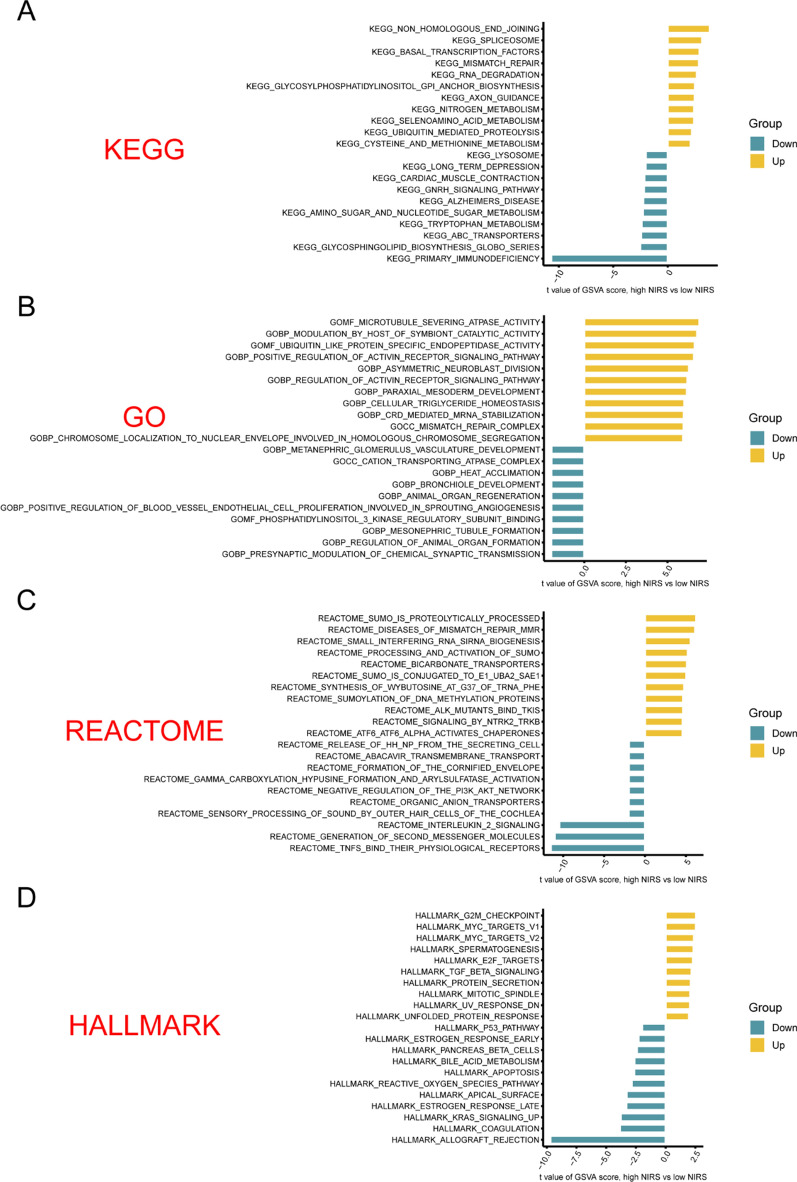
Gene set variation analysis (GSVA) of NIRS. **A** The GSVA enrichment analysis of high- and low- NIRS groups based on KEGG database. **B** The GSVA enrichment analysis of high- and low- NIRS groups based on GO database. **C** The GSVA enrichment analysis of high- and low- NIRS groups based on REACTOME database. **D** The GSVA enrichment analysis of high- and low- NIRS groups based on HALLMARK database

To unravel the potential biological functions of NIRS in UCEC, we conducted a comparative analysis of pathways exhibiting significant variation between the high- and low- NIRS groups using four sets of pathway databases: KEGG, GO, REACTOME and HALLMARK, employing the GSVA method (Fig. 12A–D). The result indicated that in the high NIRS group, compared to the low NIRS group, several pathways related to inflammatory factors, immune cell receptor signaling, and response of estrogen were significantly down-regulated. Notably, we observed a positive correlation between the high NIRS group and the MMR of genes, highlighting its potential biological significance in UCEC.

## Evaluating expression differences of three specific genes in UCEC

**Fig. 13:**
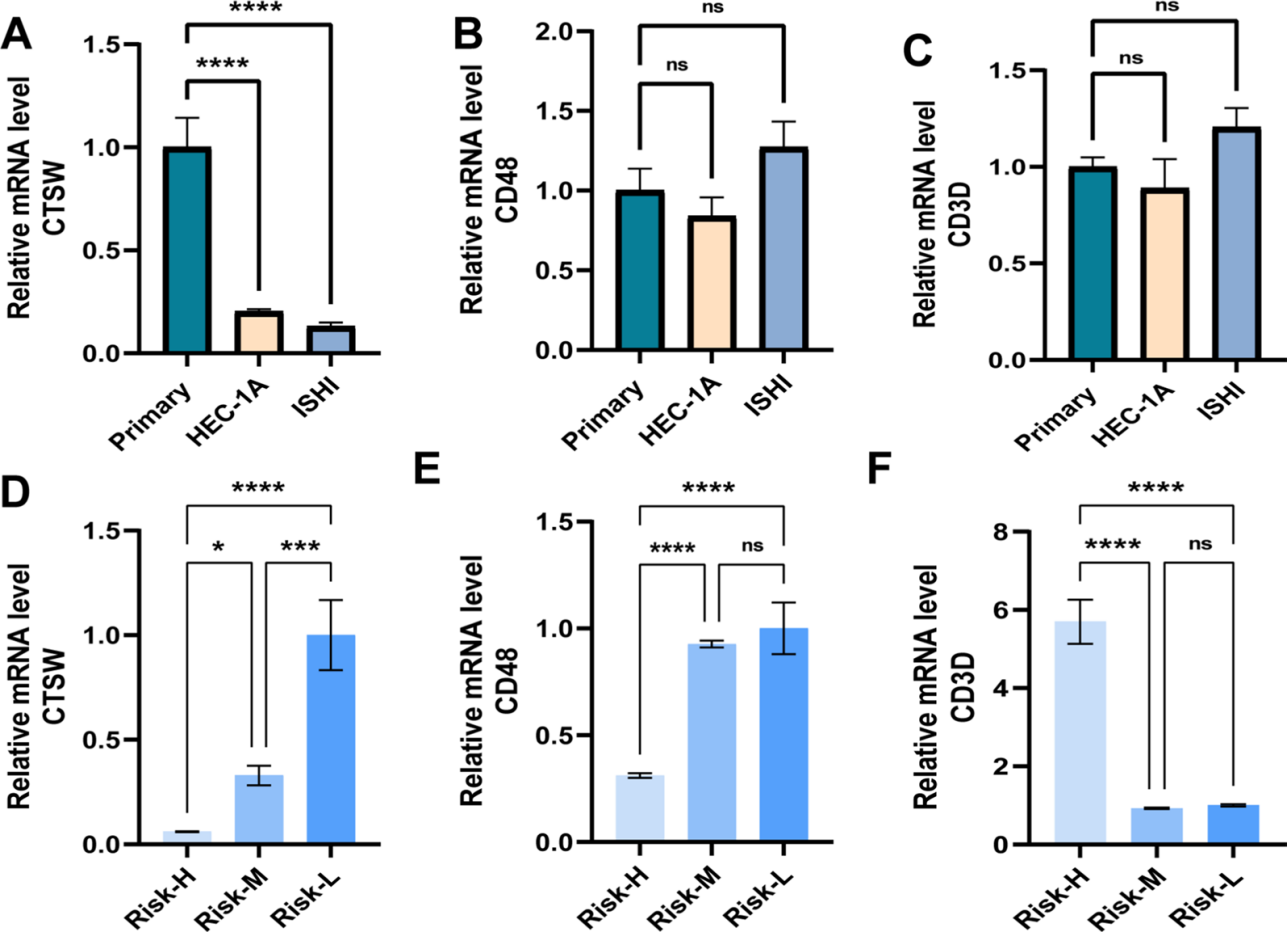
Differential expression validation of system genes. (**A**–**C**) based on qRT-PCR: The expression levels of CTSW (**A**), CD48 (**B**) and CD3D (**C**) in HEC-1A, ISHI cell lines and primary endometrial cells. (**D**–**F**) Expression results of CD48 (**D**), CD48 (**E**) and CD3D (**F**) in UCEC cancer tissue of three patients with different risk levels. Among them, ns: no significant statistical difference, *P < 0.05, **P < 0.01, ***P < 0.001, ****P < 0.0001. qRT-PCR data are means ± SD, with n = 3

To further investigate the expression disparities of the three system genes between UCEC and normal samples, we used qRT-PCR to compare their expression levels in HEC-1A, ISHI tumor cells and primary endometrial epithelial cells (Fig. 13A–C). The results demonstrated a significantly higher expression level of CTSW in primary cells compared to HEC-1A and ISHI cell lines (Fig. 13A). However, no significant difference in the expression levels of CD48 and CD3D was observed among these cell lines (Fig. 13B, D). Moreover, to validate whether the expression of these target genes varied among UCEC patients with different clinical risk profiles, we selected three UCEC cancer tissues representing low, medium, and high-risk factors for comparison using qRT-PCR (Fig. 13D–F). Intriguingly, we observed a step-like distribution of CTSW and CD48 expression from low to high in the Risk-H, Risk-M, and Risk-L groups, aligning with our previously predicted trend (Additional file 4: Figure S1A).

### Investigation of the correlation between specific genes

**Fig. 14:**
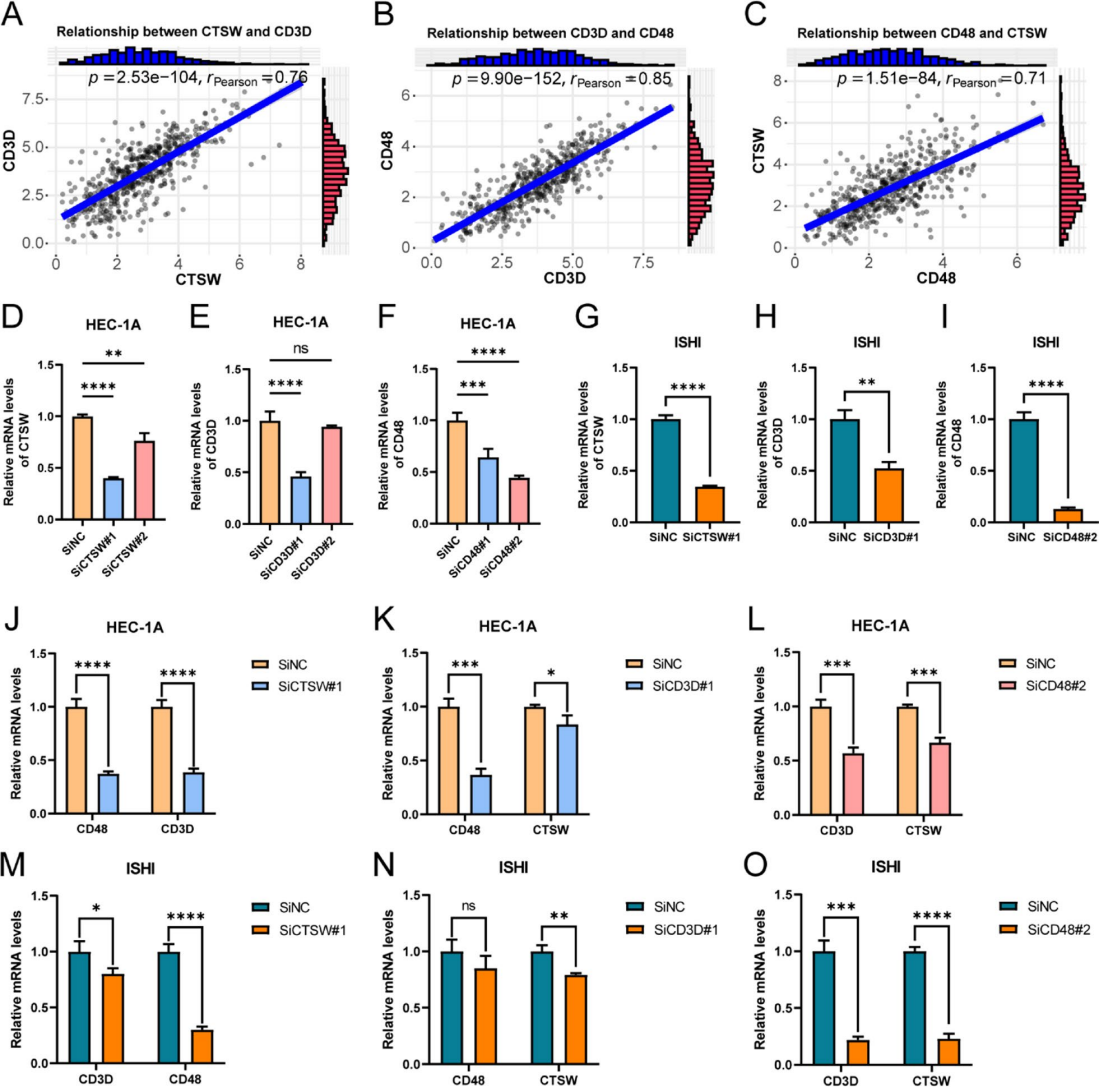
Exploration and Validation of the Interrelationships among the Three Model Genes. (**A**–**C**) Pearson correlation analysis revealed significant positive regulatory associations between CTSW and CD3D (**A**), CD3D and CD48 (**B**), as well as CD48 and CTSW (**C**) among the three model genes. (**D**–**F**) qRT-PCR validation of the designed siRNAs targeting CTSW (**D**), CD3D (**E**), and CD48 (**F**) in HEC-1A cell line, showing the downregulated expression levels of the target genes in the gene knockdown cells compared to the negative control group. (**G**–**I**) Further qRT-PCR validation of the designed siRNAs targeting CTSW (**G**), CD3D (**H**), and CD48 (**I**) in ISHI cell line, demonstrating the downregulated expression levels of the target genes in the gene knockdown cells compared to the negative control group. (**J**–**L**) The expression differences between SiCTSW (**J**), SiCD3D (**K**), and SiCD48 (**L**) and the negative control for the other two target genes. (**M**–**O**) Further qRT-PCR analysis in ISHI cell line exploring the expression differences of SiCTSW (**M**), SiCD3D (**N**), and SiCD48 (**O**) compared to the negative control for the remaining two target genes. Among them, ns: no significant statistical difference, *P < 0.05, **P < 0.01, ***P < 0.001, ****P < 0.0001. qRT-PCR data are means ± SD, with n = 3

The expression of the three selected genes (CD48, CD3D, and CTSW) showed significant correlations with the infiltration level of immune cells in the TIME of UCEC and patient prognosis in our study. To further explore potential associations between the expression levels of these three model genes, Pearson correlation analysis was conducted to examine the pairwise relationships. The results revealed significant positive correlations between CTSW and CD3D, CD3D and CD48, as well as CD48 and CTSW, with Pearson correlation coefficients of 0.76, 0.85, and 0.71, respectively (Fig. 14A–C). To further validate these findings, specific siRNAs were designed and successfully employed to downregulate the expression levels of the three target genes in HEC-1A and ISHI cells (Fig. 14D–I). Furthermore, the relative expression levels of the remaining two target genes in the gene knockdown cells were explored compared to the negative control. The results demonstrated varying degrees of inhibition in the expression levels of the other two model genes in the gene knockdown cells of HEC-1A (Fig. 14J–L) and ISHI (Fig. 14M–O) cell lines, indicating the potential interplay among these genes.

## Discussion

UCEC is one of the most prevalent malignant tumors affecting the female reproductive tract, poses a significant health burden. According to the latest data published by GLOBOCAN2020, there were 417,367 new cases of UCEC worldwide, and 97,370 cases [50] of UCEC-related deaths. The constraint of clinical treatment lies in the high heterogeneity of the tumor morphology and molecular buildup. Currently, pathological classification categorized UCEC in two main groups with distinct biological behaviors: low-grade (G1-2) and high-grade (G3) tumors [51]. In addition, the Cancer Genome Atlas (TCGA) proposed four molecular subtypes: POLE-ultramutated, mismatch repair-deficient, P53-mutated, and no-specific molecular profile endometrioid carcinoma [52–54]. Histopathological classification, based on tumor morphology and grading, plays a pivotal role in UCEC treatment as it can classify the prognosis into different risk categories and guide surgery as well as adjuvant therapy. However, tumors of the same pathological type may exhibit substantial genomic variations, leading to divergent prognoses [55]. The current evaluation methods, which rely on patient characteristics, histological type, and clinical stage, still exhibit significant limitations in accurately predicting the patients’ OS. Immunotherapy has emerged as an increasingly important treatment modality for advanced endometrial cancer. Both microsatellite status and TMB play important roles in directing the efficacy of immunotherapy and guiding clinical decision-making. However, the existing molecular typing methods cannot adequately distinguish populations with distinct immunotherapy responses, hence the establishment of more refined molecular typing is necessary. It is noteworthy that there is a large number of immune cell infiltration in UCEC tumors, and studies [56–59] have demonstrated the strong predictive value of tumor-infiltrating immune cell density in UCEC. The significant variations in T-cell infiltration among tumors may explain the differential survival outcomes observed within the same subgroups. To address this, we have developed a novel predictive model aimed at more accurately assessing patient risk stratification and treatment sensitivity in this context.

CD8^+^ T cells play a critical role in the host's immune response against tumors. They possess the ability to secrete cytotoxic molecules such as granzyme and perforin to eliminate tumor cells [60–62] as well as secrete IFN-γ to induce tumor ferroptosis [63]. It has been confirmed that the infiltration density of CD8^+^ T cells has predictive value for UCEC prognosis [23, 64, 65]. Referring to the modeling methods of published relevant research [66–69], we focused CD8^+^ T cell as the primary subject of investigation to construct a predictive system and aimed at assessing the TIME and responsiveness to immunotherapy. CIBERSORT and ssGSEA algorithms were used to evaluated the infiltration levels of immune cells in the TIME of UCEC patients. Moreover, the WGCNA algorithm explored the genes highly related to the expression of CD8^+^T cells in UCEC. NMF and random forest algorithms were used to cluster and screen the DEGs of different CD8^+^T cell-related clusters. Finally, a novel scoring system, NIRS, which is composed of CTSW, CD48 and CD3D, was constructed by univariate Cox regression, Lasso regression and multivariate Cox regression.

By assessing the comprehensive expression levels of the three target genes, we calculated the NIRS score for each patient and divided them into two cohorts of low- and high- NIRS. Survival analysis showed that patients in the low NIRS group exhibited significantly higher OS than those in the high NIRS group. ROC curve analysis and Cox regression analysis confirmed that NIRS was a highly sensitive, specific, and clinically independent prognostic risk factor. Moreover, subgroup analysis based on clinical characteristics demonstrated a significant correlation between NIRS and two conventional clinical assessment indicators, grade and stage. Patients with high NIRS were associated with a higher clinical stage and pathological grade, which was consistent with the risk factors mentioned in international guidelines [70, 71].

NIRS is a scoring system closely related to CD8^+^ T cells, and the analysis of the immune microenvironment revealed a negatively correlation between NIRS and most immune cells, while the strongest negative correlation was with CD8^+^ T cells, patients with high NIRS exhibited significantly lower levels of CD8^+^T cell infiltration and ESTIAMTE scores. All the above findings suggest that a high NIRS score may represent a lower degree of immune infiltration, especially CD8^+^T cells. Additionally, NIRS holds the potential to guide treatment selection for UCEC patients. The results demonstrated that NIRS was negatively associated with the expression levels of six common immune checkpoints: PD-1, PD-L1, CTLA-4, LAG-3, TIM-3 and TIGIT. In addition, the four different IPS in the TCIA database showed greater sensitivity in the low NIRS group, suggesting that patients with low NIRS may have higher benefits from PD-1 and CTLA-4 inhibitors. The immunotherapy cohorts used for external validation further confirmed the efficacy of NIRS in determining immunotherapy responsiveness. Across the three external validation sets, patients in the low NIRS group had better responsiveness and efficacy evaluation to ICIs, and patients in the IMvigor210 and PRJEB23709 cohorts with high NIRS had lower OS and a higher proportion of patients with progression after immunotherapy; Regarding other antineoplastic drugs, we found that patients with low NIRS had better curative effect on chemotherapy drugs represented by cisplatin and docetaxel; PARP inhibitors represented by olaparib and endocrine drugs represented by tamoxifen. However, temsirolimus exhibited greater sensitivity in the high NIRS group, suggesting it may become a treatment option for patients with high NIRS. Clinicians can formulate precise treatment plans for patients based on the prediction results from the NIRS system to achieve individualized treatment for UCEC patients.

NIRS exhibits potential crosstalk with other markers for evaluating the efficacy of immunotherapy in UCEC. TMB reflects the extent of gene mutations in tumor cells, which is processed into neoantigens and presented to T cells by major histocompatibility complex proteins. Multiple studies have confirmed that TMB as a predictor of ICIs efficacy and long-term survival rates following immunotherapy [72–76]. In our study, the low-risk group showed a significant increase in TMB, while the TMB-H/NIRS-L group had the highest long-term survival rate among the four groups, indicating that NIRS combined with TMB improves predictive efficacy. Mutation analysis revealed that the high-NIRS group had a higher TP53 mutation load, which may contribute to a poor prognosis [77–79]. Alternatively, the high-NIRS group had a lower PTEN mutation load, which is considered to be one of the causes of MSI-H [80–82]. MSI defines one of the four molecular subgroups of UCEC identified by TCGA, which reflects the loss of MMR function [83]. Immunohistochemical (IHC) detection of four MMR proteins is widely used clinically for the screening for Lynch syndrome, prognostic assessment analysis and screening of ICIs beneficiaries [84–86]. UCEC patients with MSI-H/dMMR typically have stronger immunogenicity and extensive T cell infiltration, making them more responsive to immunotherapy [86–88]. Our results demonstrated a significant negative correlation between NIRS and the mutation rate of MMR proteins. Similarly, the proportion of patients with the MSS subtype was higher in the high NIRS cohort, and the comprehensive NIRS score of patients with MSS type was significantly higher than that of the MSI type. In summary, we propose NIRS as a supplementary tool to classical molecular typing and predictive markers for immunotherapy, thereby bringing more accurate assessments of immunotherapy efficacy and the immune microenvironment in UCEC patients.

The findings obtained from CIBERSORT and ssGSEA analyses showed that the expression of the three system genes was positively correlated with the infiltration of CD8^+^T cells. Survival analysis further revealed that the OS among patients with high expression of system genes was significantly increased (Additional file 1: Figure S1B–D). The above results suggest that CTSW, CD3D, and CD48 may serve as predictive markers for the prognosis and immunotherapy responsiveness of UCEC, and we observed a significant positive correlation in their expression levels in UCEC. CD3D encodes a component of the T-cell receptor/CD3 (TCR/CD3) complex, which participates in the development and signal transduction of T cells [89]. The integrity of TCR/CD3 complex is crucial for the effect and regulatory functions of peripheral T cells [90]. CD3D has been closely related to tumor prognosis, immune microenvironment and immunotherapy responsiveness. It has been confirmed that CD3D participates in regulating the expression of tumor infiltrating lymphocytes (TILs) and immune checkpoints in gastric cancer, breast cancer, bladder cancer and other diseases [91–94], highlighting its potential as a predictor of immunotherapy efficacy across various tumor types. As an adhesion and co-stimulatory molecule, CD48 activates T cells, antigen presenting cells, and granulocytes [95]. It has been reported that CD48 can be used as a prognostic marker for diseases such as lung cancer, glioma, and colon cancer [96–98]. CTSW, another system gene, is an immune-specific cysteine proteinase belonging to the papain family [99, 100]. Other members of this family have been confirmed to be secreted into the TIME by tumors or immune cells during tumorigenesis and development [101–103], making them potential drug targets. CTSW exhibits specific expression in CD8^+^ T and NK cells [104, 105]. Zhang et al. [106] found that CTSW is a candidate tumor suppressor gene of breast cancer, which has specific regulatory functions on the infiltration level and lethal activity of CD8^+^ T cells, and has a positive correlation with the survival of patients. The predictive model constructed by Chen et al. [107] found that CTSW may become a potential indicator for judging prognosis and the efficacy of immunotherapy. As relevant molecules involved in immune activation, the coordinated expression of CTSW, CD3D, and CD48 in UCEC may be attributed to their shared regulation by common transcription factors or regulatory elements, or their involvement in overlapping signaling pathways or regulatory networks, thus facilitating their correlated expression. The dysregulation of Tfh (follicular helper T) cells induced by the interaction between CD48 and CD3D has been reported as a common pathogenic mechanism underlying the co-occurrence of dilated cardiomyopathy and atrial fibrillation [108]. However, there is currently no clear exploration or elucidation of the correlation among these three characteristic genes in the field of oncology. Further investigations such as gene expression profiling, functional analysis, and analysis of regulatory elements are warranted to unravel the precise mechanisms and functional significance of the interplay among CTSW, CD3D, and CD48 in UCEC.

In our study, analysis of the TCGA database predictions revealed a significantly lower expression of CTSW in tumor cells compared to normal cells. This prediction was further confirmed by qRT-PCR experiments, which showed an up-regulated expression of CTSW in primary cells compared to HEC-1A and ISHI cells. The consistent expression trend was observed in patients with different risk factors. However, the exact mechanism by which CTSW regulates immunity in UCEC, particularly its impact on CD8 + T cell expression, remains unclear. Further research in this area holds significant value. The GSVA results indicated numerous significant differences in immune pathways between the high- and low-NIRS groups, providing potential insights for future investigations.

Lastly, our study has certain limitations that need to be acknowledged. Firstly, this study is mainly based on bioinformatical analyses, and further clinical studies are necessary to confirm the predictive power of our model, as well as further basic research to explore the molecular mechanism through which system genes affect biological functions in UCEC. In addition, several other clinical risk factors for UCEC, such as obesity, estrogen levels, and vessel carcinoma embolus, were not included in the analysis of this study, and the combination of these clinical factors may continue to optimize the predictive efficacy of NIRS.

## Conclusion

In this study, we identified three system genes based on the infiltration level of CD8^+^ T cells and constructed a novel predictive scoring system for UCEC. NIRS not only distinguished patient populations with varying prognostic risks and immunotherapy responses, but also demonstrated potential associations with microsatellite status, TMB, MMR and even alternative anti-tumor treatment options.

## Contribution to the field statement

Uterine corpus endometrial carcinoma (UCEC) is one of the most prevalent malignant tumors of the female reproductive tract. Despite various treatment options available, such as surgery, radiotherapy, chemotherapy, hormonal therapy and targeted therapy, the overall prognosis for UCEC patients remains unsatisfactory. Therefore, the development of novel methods to assess prognosis and guide therapeutic regimes is paramount. This study describes the development of a novel immune risk score (NIRS) meant to predict UCEC patient prognosis and their responsiveness to different therapeutics. We used CIBERSORT, weighted gene co-expression network analysis (WGCNA), non-negative matrix factorization (NMF), and random forest algorithms to screen the module associated with CD8^+^ T cells and key related genes. Through these analyses, we identified three genes (CTSW, CD48 and CD3D) that were closely associated with CD8^+^T cells and other immune-related processes. These genes were then utilized to construct the NIRS through multiple screening procedures. The results of our study revealed that NIRS was closely associated with the infiltration level of many kinds of immune cells, especially CD8^+^T cells, and the expression of multiple immune checkpoints. In addition, the scoring system efficiently predicts patient’s responsiveness to different immune checkpoint inhibitor therapeutics as well. NIRS not only distinguished populations with different prognostic risks and immunotherapy responses, but also showed potential associations with microsatellite status, TMB, dMMR and alternative treatment options, providing valuable insights for personalized therapeutic strategies in UCEC.

## Supplementary Information

**Additional file 1: Table S1.** Media and related information for cell culture.

**Additional file 2: Table S2.** Summary of SiRNA sequences information.

**Additional file 3: Table S3.** Information of primer sequence in qRT-PCR sequence.

**Additional file 4: Figure S1.** Differential expression and prognosis analyses of system genes.

## Data Availability

The datasets presented in this study can be found in online repositories. The names of the repository/repositories and accession number(s) can be found in the article. The complete original data and code can be downloaded from: https://www.jianguoyun.com/p/DZwEyekQ3rToChiarYkFIAA.

## Abbreviations

CR: Complete response
DEGs: Differentially expressed genes
dMMR: Different mismatch repair
ESTIMATE: Estimation of stromal and immune cells in malignant tumors using expression data
FPKM: Fragments per kilobase million
GDCS: Genomics of Drug Sensitivity in Cancer
GO: Gene Ontology
GSVA: Gene set variation analysis
IC_50_: Half maximal inhibitory concentration
ICI: Immune checkpoint inhibitor
IHC: Immunohistochemical
IPS: Immunophenoscore
KEGG: Kyoto Encyclopedia of Genes and Genomes
K-M: Kaplan–Meier
LASSO: Least absolute shrinkage and selection operator
MMR: Mismach repair
MSI: Microsatellite instability
MSI-H: Microsatellite instability-high
NIRS: Novel immune risk score
NMF: Non-negative matrix factorization
ORR: Objective response rate
OS: Overall survival
PCA: Principal component analysis
PD: Progressive disease
PR: Partial response
qRT-PCR: Quantitative reverse transcription polymerase chain reaction
ROC: Receiver operating characteristic
SD: Stable disease
TCGA: The Cancer Genome Atlas
TCIA: The Cancer Immunome Atlas
TCR/CD3: Complex T-cell receptor/CD3 complex
TIDE: Tumor Immune Dysfunction and Exclusion
TIL: Tumor infiltrating lymphocyte
TIME: Tumor immune microenvironment
TMB: Tumor mutation burden
TOM: Topological overlap matrix
TPM: Transcripts per million
tSNE: T-distributed stochastic neighbor embedding
UCEC: Uterine corpus endometrial carcinoma
WGCNA: Weighted gene co-expression network analysis
