# Supplementary material for: Construction of the novel immune risk scoring system related to CD8+ T cells in uterine corpus endometrial carcinoma
Source: Cancer Cell Int. 2023 Jun 22;23:124. doi: 10.1186/s12935-023-02966-y (PMC10286354; doi:10.1186/s12935-023-02966-y)
Supplement: Supplementary file 1 — Additional file 1: Table S1. Media and related information for cell culture. [file 12935_2023_2966_MOESM1_ESM.docx]

**Table S1** **|** Media and related information for cell culture.

| Cell line | Medium | Manufacturer |
| --- | --- | --- |
| HEC-1A | McCoy’s 5A | Procell Life Technology (Wuhan, China) |
| ISHI | Dulbecco’s modified Eagle medium (DMEM) | Procell Life Technology (Wuhan, China) |
| Primary cell | basic medium for primary epithelial cells with 1% growth factor | Pricells Biotechnology & Medicine Co., LTD (Wuhan, China) |
