# Supplementary material for: Construction of the novel immune risk scoring system related to CD8+ T cells in uterine corpus endometrial carcinoma
Source: Cancer Cell Int. 2023 Jun 22;23:124. doi: 10.1186/s12935-023-02966-y (PMC10286354; doi:10.1186/s12935-023-02966-y)
Supplement: Supplementary file 2 — Additional file 2: Table S2. Summary of SiRNA sequences information. [file 12935_2023_2966_MOESM2_ESM.docx]

**Table S2** **|** Summary of SiRNA sequences information.

| Gene | Target sequence | Sense (5’-3’) | Antisense (5’-3’) |
| --- | --- | --- | --- |
| SiCD48#1 | CCCGGTCCTTTGGAGTAGAAT | CCCGGUCCUUUGGAGUAGAAUTT | AUUCUACUCCAAAGGACCGGGTT |
| SiCD48#2 | GCGAGTCTGTAAACTACACCT | GCGAGUCUGUAAACUACACCUTT | AGGUGUAGUUUACAGACUCGCTT |
| SiCD3D#1 | CCGTGCAAGTTCATTATCGAA | CCGUGCAAGUUCAUUAUCGAATT | UUCGAUAAUGAACUUGCACGGTT |
| SiCD3D#2 | GAGGACAGAGTGTTTGTGAAT | GAGGACAGAGUGUUUGUGAAUTT | AUUCACAAACACUCUGUCCUCTT |
| SiCTSW#1 | ATCCAGTTCAACCGGAGTTAC | AUCCAGUUCAACCGGAGUUACTT | GUAACUCCGGUUGAACUGGAUTT |
| SiCTSW#2 | CAGAACAACGAGCACAGAATT | CAGAACAACGAGCACAGAAUUTT | AAUUCUGUGCUCGUUGUUCTUTT |
| SiNC | CGCTGCAATGTCTATATGCAA | CGCUGCAAUGUCUAUAUGCAATT | UUGCAUAUAGACAUUGCAGCGTT |
