# Supplementary material for: Construction of the novel immune risk scoring system related to CD8+ T cells in uterine corpus endometrial carcinoma
Source: Cancer Cell Int. 2023 Jun 22;23:124. doi: 10.1186/s12935-023-02966-y (PMC10286354; doi:10.1186/s12935-023-02966-y)
Supplement: Supplementary file 3 — Additional file 3: Table S3. Information of primer sequence in qRT-PCR sequence. [file 12935_2023_2966_MOESM3_ESM.docx]

**Table S3** **|** Information of primer sequence in qRT-PCR sequence.

| Gene | Forward primer | Reverse primer |
| --- | --- | --- |
| GAPDH | CCCTTCATTGACCTCAACTACATG | TGGGATTTCCATTGATGACAAGC |
| CD48 | AGCTGCAAGTGCTTGACCC | CAGACTCGCCAGGTATCACAC |
| CD3D | CGTGGCTACCCTTCTCTCG | TCGTTCCCTCTACCCATGTGA |
| CTSW | CCAGCTCTATGGCTATCGGAG | GGTCCTTGATGGGTGAGATGG |
