# Supplementary figures and images for: Construction of the novel immune risk scoring system related to CD8+ T cells in uterine corpus endometrial carcinoma
Source: Cancer Cell Int. 2023 Jun 22;23:124. doi: 10.1186/s12935-023-02966-y (PMC10286354; doi:10.1186/s12935-023-02966-y)

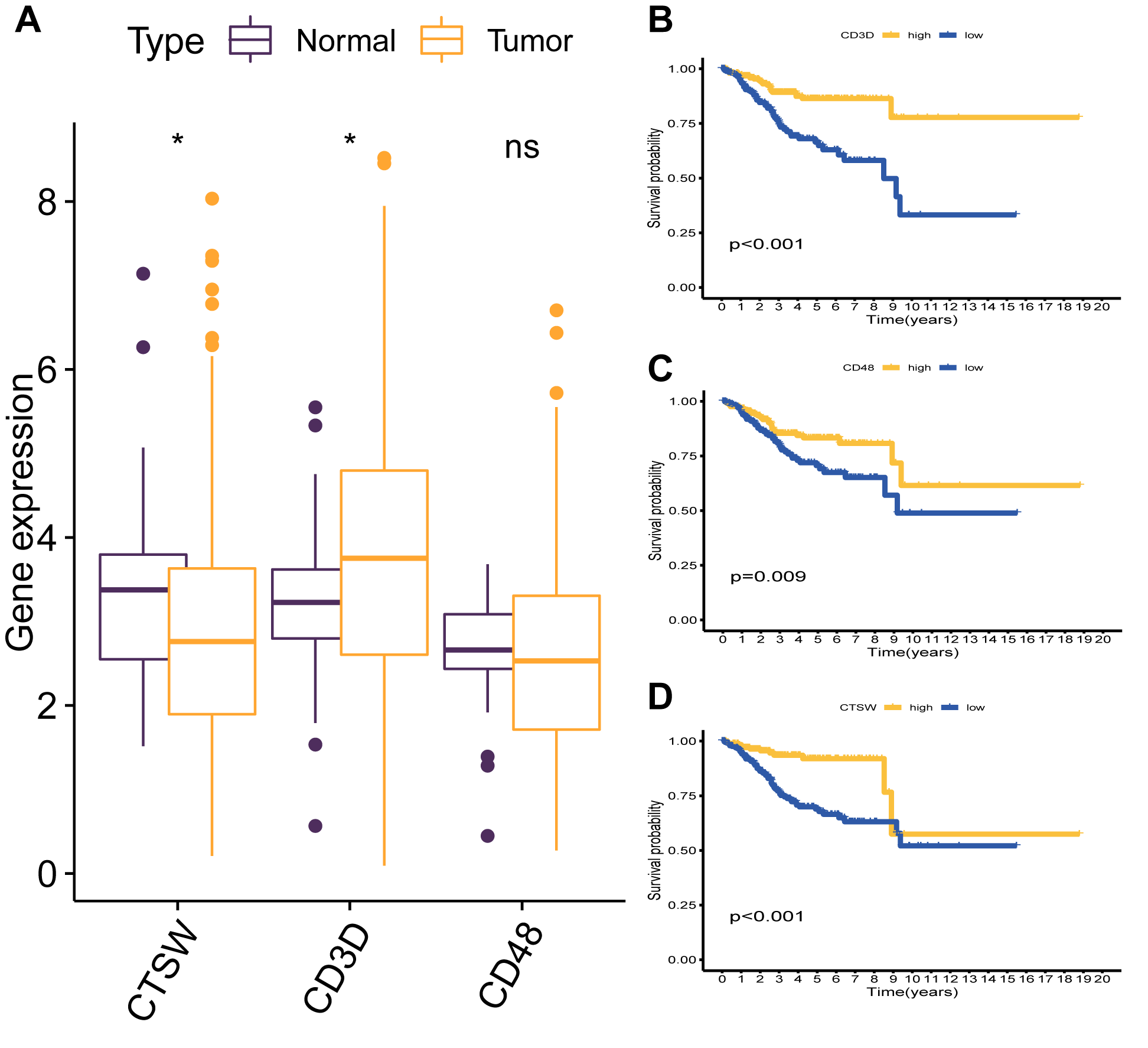

Supplement: Supplementary file 4 — Additional file 4: Figure S1. Differential expression and prognosis analyses of system genes. [file 12935_2023_2966_MOESM4_ESM.tif]
